# Clinical prediction rules for cognitive outcomes post-stroke: an updated systematic review and meta-analysis

**DOI:** 10.1016/j.eclinm.2025.103664

**Published:** 2025-11-25

**Authors:** Eugene Yee Hing Tang, Jacob Brain, Rhiannon De Ivey, Serena Sabatini, Felicity Mills, Emma Jackson, Linda Errington, Claire Burley, Jennifer Dunne, Leanne Greene, Ram Bajpai, Christopher Price, Louise Robinson, Nele Demeyere, Blossom Christa Maree Stephan, Maree Stephan, Terry Quinn

**Affiliations:** a Population Health Sciences Institute, Newcastle University, United Kingdom; b Institute of Mental Health, School of Medicine, University of Nottingham, Innovation Park, Jubilee Campus, Nottingham, United Kingdom; c Freemasons Foundation Centre for Men's Health, Discipline of Medicine, School of Psychology, The University of Adelaide, Adelaide, SA, Australia; d Department of Clinical Psychology and Psychobiology, University of Barcelona, Barcelona, Spain; e School of Psychology, University of Surrey, Guildford, United Kingdom; f School of Medicine, Dentistry and Nursing, University of Glasgow, Glasgow Clinical Research Facility, Institute of Neurological Sciences, Queen Elizabeth University Hospital, Scotland, United Kingdom; g Dementia Centre of Excellence, enAble Institute, Curtin University, Bentley, WA, Australia; h University of Exeter Medical School, University of Exeter, Exeter, United Kingdom; i School of Medicine, Keele University, Keele, United Kingdom; j Nuffield Department of Clinical Neurosciences, University of Oxford, United Kingdom; k Cardiovascular and Metabolic Health, University of Glasgow, Scotland, United Kingdom

**Keywords:** Stroke, Dementia, Risk prediction

## Abstract

**Background:**

Survivors of stroke are at a higher risk of cognitive syndromes, including dementia and delirium. Timely identification of those at-risk for cognitive syndromes could ensure better clinical management and implementation of risk reduction strategies. This study updates and appraises current evidence on prognostic accuracy of multicomponent risk models for post-stroke cognitive syndromes.

**Methods:**

In this updated systematic review, we searched multidisciplinary electronic databases between November 2019 and October 2024 for relevant studies. An updated search was conducted on May 30, 2025. Studies were included if they described a multicomponent risk prediction tool developed in a stroke population (aged ≥18 years), free of cognitive impairment/dementia at baseline, with no exclusions on language. All study designs of primary research were eligible provided the study reported a multicomponent model at any point to predict participant cognitive outcomes i.e., incident cognitive impairment, dementia or delirium. Multicomponent refers to having more than one feature in the model e.g. if the study only reported the discriminatory accuracy of a cognitive score this was not eligible. All studies had to report sufficient discriminative performance metrics to assess model performance. Data were extracted from selected studies using a pre-specified proforma. Risk of bias was assessed using the Prediction model Risk of Bias Assessment Tool (PROBAST), certainty of evidence by GRADE, and between-study heterogeneity via *I*-squared (*I*^*2*^) statistics. Our study was preregistered with PROSPERO (CRD42024601845).

**Findings:**

From 16,259 articles, 20 new studies contributed 31 models for post-stroke cognitive impairment and/or dementia and six models for post-stroke delirium with most developed in Asia (n = 12). Most models (n = 10) used logistic regression, with some using machine learning methods (n = 5). Development cohorts were small (mean n = 677). The pooled c-statistic for post-stroke cognitive impairment and delirium were 0.81 (95% CI 0.77–0.85, *I*^*2*^ 95.7%) and 0.85 (95% CI 0.77–0.93, *I*^*2*^ 52.7%), respectively. Three models externally validated (C-statistic: 0.72–0.91); and two models underwent temporal validation (AUC 0.81–0.82). Eight studies included measures of calibration which all demonstrated good calibration. Most studies (n = 17) were deemed to have low risk of bias and applicability concerns but overall certainty of evidence by GRADE was low.

**Interpretation:**

Development of risk models to predict cognitive syndromes post-stroke has increased. Development cohorts remain small, largely developed in Asia with very few assessing model transportability. Future studies should pool data and utilise the potential of routinely collected large datasets. Stakeholder engagement and cost-effectiveness of risk-stratified interventions are needed prior to clinical implementation.

**Funding:**

National Institute for Health and Care Research Advanced Fellowship.

**Research in context**

**Evidence before this study**

International guidelines recommend the development of robust methods to identify future dementia risk so that they can be stratified to future interventions. Since the first review of multicomponent risk prediction scores (n = 11) to predict cognitive syndromes in stroke was published in 2021, there has been significant momentum and research in this field, particularly in Asia. Systematic reviews that coherently bring together and appraise the evidence in this field are difficult due to the heterogeneity across studies. In this systematic review, we update the evidence base and bring uniformity to this field.

**Added value of this study**

In this updated systematic review, we searched multidisciplinary electronic databases between November 2019 to October 2024 for relevant studies, with a search update on May 30, 2025. 20 new studies contributed 31 models for post-stroke cognitive impairment and dementia plus six models for post-stroke delirium, with more advanced modelling techniques beyond traditional Cox or Logistic regression modelling being employed, such as machine learning. While models incorporate evidenced-based features such as age, education, stroke severity, diabetes and white matter hyperintensities, many include numerous risk factors that have not been proven to have prognostic utility in other studies. Despite recommendations from the previous review to use best practice guidelines to develop the models, very few assessed transportability through external (n = 3) or temporal (n = 2) validation and the datasets used were generally small (mean, n = 677), with the predominance of Asian developed models reducing generalisability to other settings. Overall certainty of the evidence was also low as assessed by GRADE.

**Implications of all the available evidence**

Our findings show that no current models to predict cognitive syndromes post-stroke can be recommended for clinical use due to developmental limitations, particularly the lack of external validation, small sample sizes and lack of certainty of the current evidence base. Whilst more advanced statistical methods are being employed, tools must be clinically interpretable and utilise features that are evidence-based for post-stroke cognitive syndromes. Harmonising cohorts or utilising electronic health records, alongside innovative methods to identify risk factors, could advance this field by revealing non-traditional relationships and enhancing the understanding of the complex interplay between known and not yet known risk factors for post-stroke cognitive difficulties. There is still a substantial gap between development of risk models and subsequent clinical implementation which needs to be addressed. Future research needs to consider the cost-effectiveness of models, intervention development to reduce risk and key stakeholder engagement prior to their adoption.

## Introduction

Stroke-survivors frequently report multiple clinical and social needs which often remain unmet long after their stroke.^1^ These unmet needs include less visible deficits in areas such as cognition, fatigue and emotional wellbeing.^1^ Until cognitive deficits are identified, gaps in patient care and post-stroke sequelae will continue to impact patients and their families^2^ due to the associations between general cognitive impairment and activity limitations and participation restrictions.^3^

Post-stroke cognitive impairment (PSCI) is common in the first-year post-stroke^4^, ^5^, ^6^ with domain-specific impairments in memory, attention and executive function being most severely and often affected.^7^ Incidence of dementia is nearly 50 times higher than the general population in the year following a major stroke.^8^ Although there are often improvements in domain-specific cognitive deficits in the first months^9^ and long term after stroke,^10^ global cognitive decline is common in the first year and beyond.^4^ Approximately 4 in 10 stroke survivors will have PSCI (no dementia),^11^ and 1 in 10 stroke-survivors develop dementia soon after their first stroke.^12^ PSCI can also persist in the long term,^13^ even in those strokes considered to be “minor”.^14^ PSCI is also associated with the long term risks of mortality and recurrent stroke^15^ as well as dependency, depression and care-home admission.^16^ In addition, delirium is also common post-stroke and an under-recognised contributor to cognitive impairment in older adults.^17^ As an independent risk factor for dementia, preventing or minimising delirium could mitigate long-term cognitive decline.^18^ Early identification of individuals at risk of post-stroke cognitive syndromes could facilitate timely support for stroke-survivors, families, and caregivers. Additionally, recognising at-risk groups could enable stratification for targeted, risk-reduction interventions, which is recommended in international guidance.^19^

A previous review in 2019 identified 11 prognostic models, seven for PSCI and four for delirium.^20^ Recommendations on their use was limited by high risk of bias and lack of evidence for transportability e.g. external validation.^20^ Recent attempts to update the literature have either included models that used machine learning^21^ or did not exclude studies where stroke-survivors may have had cognitive impairment at baseline.^22^ Exclusion of baseline cognitive impairment and dementia prior to a stroke is key to ensuring model comparability and reducing bias. Since 2019, there have been significant methodological advances to prediction model development as well as a substantial increase in the models being developed particularly in the general population for dementia prediction.^23^ With the increased emphasis on identifying those at-risk with risk reduction strategies being advocated for dementia as a whole, it is important that a synthesis of the literature is conducted in the context of stroke.

We aimed to update the original systematic review^20^ to identify, describe and appraise contemporary literature and the certainty of current evidence on prediction models for PSCI and post-stroke delirium. This review will bring together the findings of the original systematic review to provide a comprehensive overview of the features used in these models and the current state of the evidence.

## Methods

### Study design and ethics

An updated systematic review was conducted and reported in alignment with the Preferred Reporting Items for Systematic Reviews and Meta-Analyses (PRISMA) guidelines.^24^ Given this is a systematic review and meta-analysis, no ethical approval or informed consent was required for this work.

### Search strategy and selection criteria

An information specialist (LE) ran the search. Title and abstract screening were performed on Rayyan by at least 2 authors (RDI, SS, FM, EJ). Full text review was conducted using Covidence systematic review software by at least 2 authors (RDI, SS, FM, EJ, JD, CB, LG, JB). The review was registered with PROSPERO (ID: CRD42024601845).^25^

The following databases were searched: MEDLINE (Ovid), EMBASE (Ovid), PsycINFO (Ovid), CINAHL (EBSCO) and The Cochrane Library. See Supplementary Material for the search terms used. The previous review had completed their search up to Nov 13, 2019. In this update we conducted a search from the last search month (November 2019) to Oct 15, 2024 to ensure no relevant studies were omitted. For all databases, the search terms included those relevant to stroke, cognition and prognosis. An updated search was conducted on the May 30, 2025.

Studies were eligible if they included a) participants who were aged 18 or over, b) people with a clinical diagnosis of stroke and c) undertook assessments of cognitive status for PSCI, post-stroke dementia or post-stroke delirium in people free of dementia/cognitive impairment at baseline pre-stroke. There was no restriction on length of follow-up interval and cognitive recovery studies were excluded. Studies that included pre-morbid cognitive impairment and those that did not specify whether the population included those with baseline cognitive impairment or dementia were excluded to ensure homogeneity in our final selection of studies. All study designs of primary research were eligible provided the study reported a multicomponent model at any point to predict participant cognitive outcomes i.e., incident cognitive impairment, dementia or delirium. Multicomponent refers to having more than one feature in the model e.g. if the study only reported the discriminatory accuracy of a cognitive score this was not eligible. All studies had to report sufficient discriminative performance metrics to assess model performance. We excluded studies that a) involved participants who had subarachnoid haemorrhage; b) predicted performance on a single cognitive domain only (e.g. language); and c) did not have results available in a full published paper in a peer-reviewed journal e.g. conference abstracts. No restrictions were placed on study setting, length of time from index stroke to follow-up or language.

### Data extraction, quality assessment and certainty of evidence

One author (EYHT) used a pre-specified proforma to extract data from the included studies which was verified by another author (JB). This included information on: study setting and design, sample characteristics, predictors/features and outcome variables, methods of model derivation, validation and measures of prediction rule performance including discrimination and calibration. Validation was further grouped by the type of validation performed e.g. internal, external or temporal validation^26^ where temporal validation uses the same study setting but participants sampled at a different time point. Risk of bias was assessed by the Prediction model Risk of Bias Assessment Tool (PROBAST).^27^ The tool consists of four domains: participants, predictors, outcome and analysis. Each domain is appraised separately and then considered together to make an overall judgement on risk of bias. Further, three study domains: participants, predictors and outcome are rated on applicability i.e., the relevance to the populations and settings that the study targets.

We also used GRADE (Grading of Recommendations, Assessment, Development, and Evaluations) (TQ) to evaluate the certainty of the overall body of evidence across both reviews. We appraised the limitations due to risk of bias, inconsistency, imprecision, indirectness and publication bias.

### Assessment of features

To harmonise all known risk variables in all risk prediction models for PSCI and post-stroke delirium, we ensured this current update followed the same framework and guidance as the original review.^20^ We then categorised the known features across all models to provide an overview and appraise the features that are currently being used in this field.

### Post-Hoc data synthesis

Results were narratively summarised using descriptive measures such as frequencies and percentages for categorical variables and mean and SD (or median and interquartile range [IQR]) for continuous variables.

The retrieved discrimination measure (i.e., c-index, or area under the receiver operating characteristics [ROC] curve, AUROC) for a developed model was summarised into a weighted average. For each study, we identified the main or recommended model, and average estimate if multiple models are fitted without any preferred model. For any c-statistic, if 95% confidence interval (CI) was not reported then we estimated it using the observed events and sample size as suggested by Debray et al.^28^ In meta-analysis, we separately pooled reported c-indices from prediction models when developed for post-stroke cognitive impairment including dementia, or delirium. We used random-effects model with restricted maximum likelihood (REML) estimation for pooled estimate, and the Hartung-Knapp-Sidik-Jonkman (HKSJ) method to calculate its 95% CIs.^29^ The proportion of variability in c-indices due to the between-study heterogeneity was summarised using *I*-squared (*I*^*2*^) statistics (*I*^*2*^ ≤ 25% for low, *I*^*2*^ < 50% for moderate, *I*^*2*^ ≥ 50% for substantial).^30^ Further, a 95% prediction interval for the random-effects model was also reported to understand the possible range of c-statistic if a new model is fitted.^31^ Publication bias was assessed by funnel plot (if there are at least 10 studies for a given outcome), and its asymmetry was tested by Egger's linear regression method (p < 0.1 was considered significant). A subgroup analysis for choice of modelling approach (regressed-based versus machine learning) was also conducted to understand heterogeneity across c-indices. We further used univariate meta-regression to explore potential variation due to the age of participants, study sample size, follow-up time, and number of observed events using the random-effects model with REML estimation. All statistical analyses were performed using Stata v19.5 (StataCorp, College Station, Texas, USA) using “*metan*” package and “*meta regress*” command.

### Role of the funding source

The funders had no involvement in study design, data collection, data analyses, data interpretation, or the writing of the report.

## Results

### Summary of included studies

**Fig. 1:**
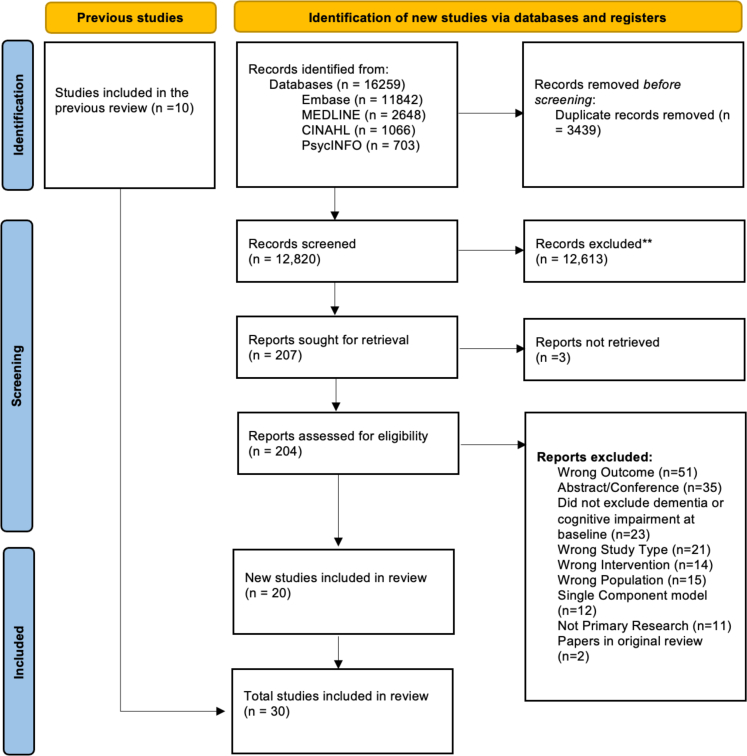
**PRISMA 2020 flow diagram for updated systematic reviews**.

From 16,259 articles of the original search, 20 studies met the inclusion criteria, of which 17 studies (n = 31 models with unique features) were for PSCI^32^, ^33^, ^34^, ^35^, ^36^, ^37^, ^38^, ^39^, ^40^, ^41^, ^42^, ^43^, ^44^, ^45^, ^46^, ^47^, ^48^ and three studies (n = 6 models with unique features) for post-stroke delirium were identified^49^, ^50^, ^51^ (Fig. 1). The updated search, performed on the 30th May 2025 yielded a further 12 studies that were eligible for inclusion. These are reported separately (Supplementary Table S4) and were not included in the analysis. The majority of models were developed in Asia (n = 12) (China (n = 9),^35^^,^^38^^,^^40^^,^^42^^,^^44^, ^45^, ^46^, ^47^, ^48^ Thailand (n = 1),^36^ South Korea (n = 1)^41^ and Taiwan (n = 1). One study harmonised cohorts from France, Germany, Australia and the United Kingdom.^34^ The sample size for PSCI ranged from 104^48^ to 3741^33^ with a sample size range for post-stroke delirium between 102^50^–514^49^ (mean for development cohorts across both, n = 677). The proportion of the cohort which developed PSCI/dementia ranged from 10%^39^ to 64%.^48^ The proportion of outcomes ranged from 29%^51^ to 50%^50^ for delirium. Characteristics of the included studies are described in Table 1.

**Table 1:** Characteristics of included studies.

Study	Country	Setting	Design	Stroke type	Development sample size, *N*	Mean age
**Post-stroke cognitive impairment**						
*Cox regression*						
Ashburner 2024^33^	US	Primary Care Practice-Based Research Network at Massachusetts General Hospital	Retrospective cohort	Ischaemic stroke	3741	71.4 years (SD: 11.8)
Molad 2019^43^	Israel	Department of Emergency Medicine at Tel-Aviv Medical Centre; Tel-Aviv Brain Acute Stroke Cohort (TABASCO)	Prospective cohort	Mild/moderate first acute ischemic stroke or transient ischemic attack	397	66.9 ± 9.7 years
*Logistic regression*						
Chu 2023^35^	China	Minhang Hospital of Fudan University	Prospective cohort	Acute ischaemic stroke	1342	68 years
Dharmasaroja 2022^36^	Thailand	Thammasat University Hospital	Prospective cohort	Ischaemic stroke	177	Non-dementia mean age 61.7 years, vascular dementia mean age 74.5 years
Georgakis 2023^37^	Germany	Multicentre hospital-based cohort study across 7 tertiary stroke centres	Prospective cohort	Acute stroke	666 in total sample	67.9 years
Gong 2021^38^	China	Stroke centre	Prospective cohort	Acute ischaemic stroke	228	62.16 years
Huang 2022^40^	China	Second Affiliated Hospital of Guangzhou Medical University and the Second People's Hospital of Foshan	Prospective cohort	Ischaemic stroke	368	71
Lee 2023^41^	South Korea	Tertiary academic hospital	Retrospective cohort	Acute ischaemic stroke	951	65.7 ± 11.9 years
Ma 2022^42^	China	Department of Neurology	Prospective cohort	Acute ischaemic stroke in diabetics	161	**No overall cohort data** No cognitive impairment: 65 years Mild cognitive impairment: 68 years Severe cognitive impairment: 74 years
Pan 2023^44^	China	Tongji Hospital, Wuhan First Hospital, and Wuhan Central Hospital in Wuhan City, Hubei Province	Prospective cohort	Acute ischaemic stroke	676	60 years
Wang 2024^45^	China	Neurology Department (Forst Hospital of Jilin University)	Prospective cohort	Acute mild ischemic stroke	285	62.3 years
Zhao 2024^47^	China	First Hospital of Jilin University	Prospective cohort	Acute minor ischemic stroke and TIA	224	61 years
*Machine learning*						
Aamodt 2021^32^	Norway	Five Norwegian hospitals (Nor-COAST)	Prospective cohort	Acute ischaemic or haemorrhagic stroke	203	Not specifically stated for this subgroup but overall cohort was 71.7 years
Betrouni 2022^34^	Harmonised cohorts—STROKOG (France, Germany, Australia, United Kingdom)	STROKDEM, DEDEMAS, Sydney Stroke Study, STRATEGIC	Prospective cohort	Acute stroke	327	STROKDEM 64.09 DEDMAS 70.25 Sydney Stroke Study 72.01 STRATEGIC 69.47
Hasan 2024^39^	Taiwan	Taipei Medical University: Taipei Medical University Hospital (TMUH), Wanfang Hospitals, and Shuang-Ho Hospital.	Retrospective cohort	Stroke	2234 (n = 1787 for training and 447 for testing)	Shuang-Ho = 65.88 TMUH = 68.77 Wangfang = 69.23
Yuan 2021^46^	China	First Affiliated Hospital of Jinzhou Medical University	Prospective cohort	Ischaemic stroke	376	PSCI 67.88 years Non PSCI 65.32 years
Zhu 2020^48^	China	Hospital-based	Prospective cohort	Ischaemic stroke	104	64.0 years
**Post-stroke delirium**						
Guldolf 2021^49^	Belgium	Stroke Unit of the University Hospital of Brussels	Prospective cohort	Acute ischaemic stroke	514	Delirium 82 years No Delirium 71 years
Haight 2020^50^	USA	Neurosciences Intensive Care Unit at a large, urban, Comprehensive Stroke Centre in Baltimore, Maryland	Prospective cohort	Acute cerebral infarct or primary intracranial haemorrhage	102	65 years
Klimiec-Moskal 2022^51^	Poland	Single-centre, hospital-based study carried out in the Department of Neurology, University Hospital, Krakow, Poland	Prospective cohort	Ischaemic stroke or TIA or Intracerebral haemorrhage	459	Median 73 years

### Prediction scores for post-stroke cognitive impairment including dementia

Most studies (n = 15) focused on prediction of cognitive impairment,^32^^,^^34^^,^^35^^,^^37^, ^38^, ^39^, ^40^, ^41^, ^42^, ^43^, ^44^, ^45^, ^46^, ^47^, ^48^ with one study predicting PSCI or dementia^33^ and another predicting vascular dementia.^36^ All 17 studies included a statement regarding excluding dementia or cognitive impairment at baseline either from the paper itself or in reference to the original cohort used. From the 17, 5 studies mentioned specific assessment as to how they excluded pre-morbid cognitive impairment including the use of Diagnostic and Statistical Manual of Mental Disorders IV criteria,^32^^,^^43^ Informant Questionnaire on Cognitive Decline in the Elderly (IQCODE).^37^^,^^40^^,^^48^ For example, *Huang* et al. states that the IQCODE was used to rule out other confounding factors such as pre-stroke cognitive function.^40^ In total there were 31 new models which used unique features rather than assessing the same features with different statistical analysis. All studies explicitly stated that individuals with pre-existing cognitive impairment and/or dementia were excluded. The main statistical methodology used was logistic regression (n = 10),^35^, ^36^, ^37^, ^38^^,^^40^, ^41^, ^42^^,^^44^^,^^45^^,^^47^ followed by machine learning (n = 5)^32^^,^^34^^,^^39^^,^^46^^,^^48^ and then Cox regression (n = 2).^33^^,^^43^

**Fig. 2:**
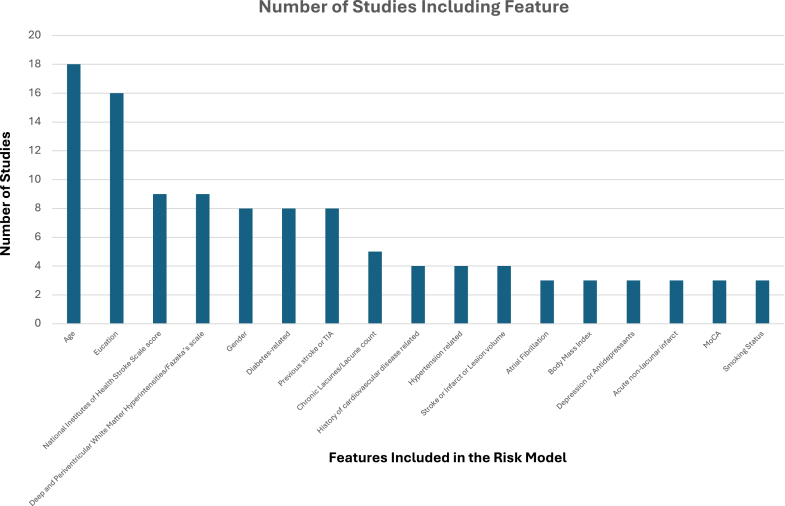
**Number of studies containing 3 or more features by category**.

Numbers of variables ranged from two^43^^,^^45^ (where the prognostic nutritional index utilises 2 blood marker features) to 30^41^ (Table 2). Across both reviews, demographic information such as age and education were the most commonly used variables (n = 22).^32^, ^33^, ^34^, ^35^, ^36^, ^37^, ^38^, ^39^, ^40^, ^41^, ^42^, ^43^, ^44^, ^45^, ^46^, ^47^, ^48^^,^^52^, ^53^, ^54^, ^55^, ^56^ Health factors were the least featured category with five models including smoking status,^37^^,^^41^^,^^43^ alcohol consumption^37^ and transfer from hospital to a facility.^33^ The previous review did not identify any models which used health factors. The next two most common categories for features were imaging (n = 17)^32^^,^^34^^,^^36^, ^37^, ^38^^,^^40^^,^^41^^,^^43^, ^44^, ^45^, ^46^, ^47^^,^^52^, ^53^, ^54^^,^^56^^,^^57^ and medical history (n = 15).^32^^,^^33^^,^^35^, ^36^, ^37^^,^^39^, ^40^, ^41^, ^42^, ^43^^,^^46^, ^47^, ^48^^,^^53^^,^^55^ For medical history, the two most common comorbidities included in the studies were diabetes (n = 8)^35^^,^^37^^,^^39^^,^^41^, ^42^, ^43^^,^^46^^,^^53^ and previous stroke or TIA (n = 8).^32^^,^^36^^,^^37^^,^^41^^,^^42^^,^^46^^,^^47^^,^^55^ Compared to the previous review where no models used any laboratory markers. In this update, seven studies included laboratory (including genetic^33^) markers in their models.^33^^,^^35^^,^^37^^,^^38^^,^^41^^,^^42^^,^^45^^,^^47^ These markers included both single value markers (e.g. fasting blood sugar,^41^ APOEe4^33^ and HbA1c^38^) and specific scores utilising blood markers such as the prognostic nutritional index,^45^ the systemic inflammatory response index^35^ and the systemic immune inflammation index.^47^ Across both reviews there were a total of 101 unique variables with imaging variables being the most frequently reported (demographics = 5, medical history = 31, symptom severity = 2, stroke type = 4, imaging = 34, laboratory markers = 13, baseline function = 9, health factors = 3) (Supplementary Table S1). Further across both reviews, the most common variables were age (n = 18) and education (n = 16) followed by stroke severity measured by the National Institutes of Health Stroke Scale score (n = 9) and variables associated with White Matter Hyperintensities (n = 9) (Fig. 2) (Table 3).

**Table 2:** Prognostic score features, outcomes and assessment.

Study	Features (n)	Outcome	Ascertainment of cognition	Timepoint of outcome assessment	Participants with outcome, N (%)	Type of model	Discrimination	Calibration	Validation
**Post-stroke cognitive impairment**									
*Cox regression*									
Ashburner 2024^33^	Full model: Age, insurance, mobility problems, prior history of falls, delirium, peripheral vascular disease, Parkinson's disease, depression, severe chronic kidney disease, abnormal weight loss and anorexia, and discharge from the hospital to a facility (n = 11) Full model minus insurance (n = 10) Full model without excluding patients with a prior history of stroke (n = 11)	Post-stroke cognitive impairment or dementia	ICD-9/10 codes	5 years	332 (11.4%) PSCI	Cox proportional hazards	Full model: C-statistic 0.750 (95% CI: 0.726–0.775); Full model minus insurance 0.749 (0.724–0.774) Full model without excluding patients with a prior history of stroke 0.750 (0.726–0.773)	None	**Internal validation** (n = 1925 (166 cases) C-statistic 0.731 (0.694–0.768); **External validation** (n = 2237 (128 cases) 0.724 (0.681–0.766)
Molad 2019^43^	Vascular (Framingham risk score for stroke (age, systolic blood pressure, antihypertensive medication, diabetes, cigarette smoking, history of cardiovascular disease, atrial fibrillation), White Matter Hyperintensity Volume, lacunes, and CMB) (n = 4) AD associated markers (APOE4 status and hippocampal volume) (n = 2)	Mild cognitive impairment	MCI (Petersen Criteria) Participants with suspected cognitive impairment were referred to an experienced cognitive neurologist. Assessments were further reviewed y a consensus forum to determine MCI versus dementia (assessor, three senior neurologists and a neuropsychologist)	2 years	80 (20.2%)–9 developed dementia and 71 developed MCI	Cox regression	Vascular related measures, AUC: 0.67 (0.56–0.78) AD related measures, AUC: 0.58 (0.45–0.67) AD and vascular related measures AUC: 0.66 (0.55–0.77)	None	None
*Logistic regression*									
Chu 2023^35^	Systemic inflammatory response index, diabetes mellitus, gender, admission NIHSS scores, education and age (n = 6)	Post-stroke cognitive impairment	MMSE	2 weeks	690 (51.4%)	Logistic regression	AUC: 0.716	1000 bootstrap resamples–good agreement was seen between the predicted risk and the observed risk in the calibration curves for this model. The Hosmer–Lemeshow test (p = 0.325) further confirmed the good calibration	None
Dharmasaroja 2022^36^	Age, education, History of stroke, white matter hyperintense lesions (Fazekas scale), stroke subtype (n = 5)	Vascular dementia	Clinical diagnosis of vascular dementia was made by senior neurologists at 6 (±1) months after the stroke based on NINDS-AIREN criteria	6 months	48 (27.1%)	Logistic regression	Cutoff point of ≥5, AUC 0.76 (0.69–0.83)	Calibration was examined by plotting predicted probability of the risk score against the actual probability of the patients who developed vascular dementia at every risk score point–the risk score showed good calibration	None
Georgakis 2023^37^	Model 1 includes age, sex, education, vascular risk factors (history of hypertension, diabetes, atrial fibrillation, prior stroke, current smoking, alcohol consumption, body mass index, circulating low-density lipoprotein cholesterol [LDL-C] levels), National Institutes of Health Stroke Scale (NIHSS) and Montreal Cognitive Assessment (MoCA) in the acute phase, pre-stroke mRS, and normalised stroke lesion volume (stroke lesion volume/total intracranial volume) n = 8) Model 2 includes the global SVD score (lacunes, white matter hyperintensities, cerebral microbleeds and enlarged perivascular spaces) + model 1 features (n = 9) Model 3 includes individual SVD markers (lacune count, deep and periventricular white matter hyperintensity (WMH) Fazekas grades, cerebral microbleed counts, and grade of perivascular spaces) + model 1 (n = 12)	Cognitive impairment	A comprehensive neuropsychological battery of tests was performed and classified in five domains (executive function, memory, language, attention, and visuospatial function)	12 months	Not specifically stated	Logistic regression	Model 1 AUC: 0.688 (0.628–0.748) Model 2 AUC: 0.701 (0.642–0.760) Model 3 AUC: 0.722 (0.664–0.779)	Overall calibration of all models was good (all Hosmer Lemeshow–derived goodness-of-fit P > 0.05)	None
Gong 2021^38^	Age, female, Fazekas Score, Educational level, number of intracranial atherosclerotic stenosis, HbA1c and cortical infarction (n = 7)	Post-stroke cognitive impairment	MoCA	6–12 months	122 (53.5%)	Logistic regression with nomogram	AUC 0.810	Calibration of the risk prediction model was assessed in the development cohort by the plot comparing the observed probability of PSCI according to the total score of the nomogram against the predicted probability based on the nomogram and by using the Hosmer–Lemeshow test that assesses whether or not the observed event rates matched the expected rates in patients with minor stroke. The calibration curve of the nomogram for the predicted probability of PSCI in patients with minor stroke demonstrated good agreement in this cohort]	**Temporal validation:** Same centre but different timepoint n = 66, AUC 0.812
Huang 2022^40^	Pre-stroke cognitive function, age, years of education, NIHSS at admission, history of ischaemic heart disease, number of chronic lacunar infarcts, medial temporal atrophy score (n = 6)	Cognitive dysfunction	MMSE	Not stated	191 (51.9%)	Logistic regression	**Training** C-index 0.846 (0.807–0.885) **Validation** n = 367 (196 (53.4%) cases) C-index: 0.845 (0.805–0.885)	Bootstrap calibration plot–good agreement between the nomogram's predictions and the actual observed cognitive impairment, indicating high predictive accuracy (mean absolute error = 0.021)	None
Lee 2023^41^	Age, Sex, Body mass index, Education years, Previous modified Rankin Scale, History of hypertension, History of diabetes mellitus History of hyperlipidemia, History of coronary heart disease, History of stroke or TIA History of atrial fibrillation, Smoking status, Discharge, NIHSS, TOAST classification. Multiple lesions Left sided lesions Stroke volume (mm3) Presence of cortical lesion Presence of subcortical lesion Presence of infratentorial lesion Presence of strategic lesion Modified Fazekas score Any chronic microbleeds Total mesial temporal lobe atrophy Fasting blood glucose Creatinine Total cholesterol Hemoglobin Systolic blood pressure, short geriatric depression scale (SGDS) (n = 30)	Post-stroke cognitive impairment	Korean Version of the Vascular Cognitive Impairment Harmonisation Standards-Neuropsychological Protocol (K-VCIHS-NP) K-MMSE MMSE-z	3–6 months	290 (30.5%)	Logistic regression Support vector machine (SVM) Extreme Gradient Boosting (XGB) Artificial Neural Network (ANN)	**K-VCIHS-NP AUCs** XGB: 0.7919 (0.6839–0.8866) ANN: 0.7365 (0.6202–0.8438) SVM: 0.7157 (0.5914–0.8271) Logistic Regression: 0.7121 (0.5914–0.8265) **MMSE-z AUCs** **XGB:** 0.7876 (0.6711–0.8892) **ANN:** 0.7339 (0.6018–0.8525) **SVM:** 0.7463 (0.6191–0.8566) **Logistic Regression:** 0.7608 (0.6434–0.8663) **MMSE AUCs** **SVM:** 0.8751 (0.7838–0.9472) **ANN:** 0.8741 (0.8165–0.9241) **Logistic regression:** 0.8713 (0.7831–0.9414) **XGB:** 0.8616 (0.7683–0.9389)	None	None
Ma 2022^42^	Sex, age, education level, recurrent cerebral infarction, course of diabetes and serum albumin (n = 6)	Post-stroke cognitive impairment	MoCA	Not stated	94 (58.39%)	Logistic regression	AUC = 0.966	None	None
Pan 2023^44^	Models without disconnection score (reference models) included 6 known predictors: age, sex, education level, baseline National Institutes of Health Stroke Scale, lesion volume, and location impact score. (n = 6) Combined model: Disconnection score (defined as the weighted sum of voxel intensities (Z score statistics) for VDSM-significant voxels that overlapped with the patient's disconnection-severity map (voxel-wise disconnection severities as weights) + Reference model (n = 7)	Post-stroke cognitive impairment	MoCA	3 months	251 (37.1%)	Logistic regression	**AUC** **Training (Reference Model)** Dataset 1: 0.738 Dataset 2: 0.741 Dataset 3: 0.732 **Training (Combined Model)** Dataset 1: 0.796 Dataset 2: 0.781 Dataset 3: 0.776 **AUC** **Testing (Reference Model)** Dataset 1: 0.700 Dataset 2: 0.657 Dataset 3: 0.694 **Testing (Combined Model)** Dataset 1: 0.740 Dataset 2: 0.710 Dataset 3: 0.755	None	None
Wang 2024^45^	Age, education, deep white matter hyperintensity (DWMH) (n = 3) Prognostic nutritional index (PNI) (serum albumin (g/L) + 5 × lymphocyte count) (n = 2) PNI as continuous variables co-diagnoses + education, stroke history and DWMH (n = 5) PNI as categorical variable co-diagnose + education, stroke history, DWMH (n = 5)	Post-stroke cognitive impairment	MMSE	6–12 months	121 (42.5%)	Logistic regression	Age, education and DWMH AUC = 73.7%; PNI as continuous variable AUC = 60.7 PNI as continuous variables co-diagnoses + education, stroke history and DWM AUC = 76.7%, PNI as categorical variable co-diagnose + education, stroke history, DWMH AUC 76.1	None	None
Zhao 2024^47^	Model 1: Sex, education level, NIHSS score, hypertension, previous stroke, deep white matter hyperintensity score (n = 6) Model 2: Model 1+ neutrophil percentages (n = 7) Model 3: Model 1+ lymphocyte percentages (n = 7) Model 4: Model 1 + neutrophil values (n = 7) Model 5: Model 1 + neutrophil-to lymphocyte ratio (n = 7) Model 6: Model 1 + systemic immune inflammation index (n = 7)	Post-stroke cognitive impairment	MoCA	6–12 months	88 (39.3%)	Logistic regression	**AUC** Model 1: 0.765 (0.702–0.827) Model 2: 0.804 (0.747–0.861) Model 3: 0.796 (0.738–0.854) Model 4: 0.78 (0.719–0.841) Model 5: 0.803 (0.745–0.86) Model 6: 0.799 (0.741–0.858)	None	None
*Machine learning*									
Aamodt 2021^32^	Stroke volume, antiplatelets, occipital th. (left), stroke severity, temporal th. (left), previous infarction, previous ICH, education (years), cingulate (right) (n = 9)	Post-stroke cognitive impairment	DSM-5 from neuropsychological test scores and instrumental activities of daily living	3 months	63 (27.8%) were categorised as having mild NCD, whereas 62 (27.3%) had major NCD	Support vector machine	AUC 0.802	None	None
Betrouni 2022^34^	Texture features kurtosis and IDM from the entorhinal cortex, and kurtosis and entropy from the hippocampus, age, and baseline MoCA score. (n = 4)	Post-stroke cognitive impairment	Overall, cognitive function was assessed by administering an extensive battery of neuropsychological tests, classified into 5 cognitive domains (memory, executive function, attention, language, and visuospatial ability).	6–12 months	STROKDEM 75 (46.9%) DEDEMAS 11 (19.6%) Sydney Stroke Study 11 (16.2%) STRATEGIC 13 (30.2%)	Random Forest	AUC 0.90 ± 0.03	None	0.77
Hasan 2024^39^	Age, disease of the circulatory system, sex, drugs related to acidity, antithrombotics, drugs related to functional gastrointestinal disorders, hypnotic, systemic use of antibacterials, NSAID, stomatological preparations, ophthalmologicals, drugs for constipation, antidepressant, analgesics, cough/cold preparations, poorly ill-defined conditions, respiratory diseases, diabetes drugs, diseases of the nervous system, antihemorrhagics (n = 20)	Post-stroke cognitive impairment	ICD-9-CM and ICD-10 codes	Stroke index date until September 30, 2017, or until their last follow-up	319 (9.9%)	CatBoost Classifier, Extreme Gradient Boosting, Light Gradient Boosting machine, Extra Tree Classifier, Random Forest Classifier	**CatBoost model:** AUC = 0.93 **XGB Classifier:** AUC = 0.92 **LGBM Classifier:** AUC = 0.92 **ExtraTrees Classifier:** AUC = 0.91 **Random Forest Classifier:** AUC = 0.92	Calibration plots demonstrated well calibrated and high performing prediction model	**External Validation:** Wanfang. Hospital (n = 975), AUC = 0.91
Yuan 2021^46^	Years of education, history of stroke, history of diabetes, left frontal NAA/Cr, left thalamus NAA/Cr and left hippocampus NAA/Cr (n = 6)	Post-stroke cognitive impairment	MMSE and MoCA	3–6 months	118 (31.3)	LASSO and Nomogram	AUC: 0.8935 (0.823–0.910)	The consistency test between the predicted and actual values through the calibration plot showed that the predicted probability of the nomogram for PSCI correlates well with the actual diagnosis.	**External Validation** 227 stroke patients (75 PSCI and 152 N–PSCI), who were hospitalised between May 2019 and September 2020 at the Third Affiliated Hospital of Jinzhou Medical University (143) and the Central Hospital (84); n = 75/227 AUC: 0.8523 (0.831–0.908)
Zhu 2020^48^	Baseline MoCA scores, education, BMI and baseline MMSE scores (n = 4)	Post-stroke cognitive impairment	MMSE and MoCA A comprehensive neuropsychological battery that evaluated four cognitive domains: (1) language (Boston Naming Test); (2) visuoconstruction (Clock Drawing Test; (3) verbal memory (Auditory Verbal Learning Test; and (4) executive function/attention (Trail Making Test. Impairment was defined by the attainment of a result that was 1.5 standard deviations below the standardised mean. The diagnosis of PSCI required deficits in at least one domain, as assessed by the neuropsychological battery.	3–6 months	66 (63.5%)	Classification and Regression Tree	AUC 82.3%	None	None
**Post-stroke delirium**									
Guldolf 2021^49^	NIHSS, age, neutrophil-lymphocyte ratio, premorbid mRS, history of previous stroke, premorbid cognitive dysfunction and hearing problems (n = 7)	Delirium	DSM-5 Criteria	7 days	201 (39%)	Logistic regression	**AUC** Age and NIHSS 0.82 (0.78–0.85) NIHSS, age and premorbid cognitive dysfunction 0.82 (0.79–0.87) NIHSS, age and NLR 0.83 (0.79–0.87) Age, NIHSS, NLR and premorbid cognitive dysfunction 0.84 (0.81–0.88) Dichotomised Models Age ≥75 and NIHSS ≥7 0.76 (0.73–0.82) NIHSS ≥7, age ≥75 years and premorbid cognitive dysfunction 0.80 (0.76–0.84) NIHSS ≥7, age ≥75 and NLR >2.50 0.80 (0.76–0.84) Age ≥75, NIHSS ≥7, NLR >2.50 and premorbid cognitive dysfunction 0.82 (0.78–0.86)	None	None
Haight 2020^50^	Age greater than 64 years, presence of intraventricular haemorrhage (IVH), intubation, presence of acute kidney injury (AKI), and stroke with either cognitive deficit, neglect, or aphasia (n = 5)	Delirium	CAM-ICU	72 h	51 (50%)	Logistic regression	AUC 0.9	None	**Temporal validation:** AUC 0.82 (n = 100)
Klimiec-Moskal 2022^51^	Model A: Age and stroke severity (NIHSS) (n = 2) Model B: Age, stroke severity (NIHSS) and C reactive protein (n = 3) Model C: stroke severity (NIHSS), atrial fibrillation, diabetes mellitus, pre-stroke dependency and haemorrhagic stroke (n = 5) Model D: stroke severity (NIHSS), atrial fibrillation, diabetes mellitus, pre-stroke dependency, haemorrhagic stroke and C reactive protein (n = 6)	Delirium	Brief Confusion Assessment Method (bCAM) for verbal patients and the Confusion Assessment Method for the Intensive Care Unit (CAM-ICU) for non-verbal patients	7 days	134 (29.2%)	Logistic regression	**AUC** Model A: 0.77 (0.71–0.81) Model B: 0.80 (0.76–0.84) Model C: 0.81 (0.77–0.85) Model D: 0.84 (0.80–0.88)	Both models were well-calibrated as assessed by the Hosmer–Lemeshow test (p = 0.532 for Model A and p = 0.253 for Model B).	None

**Table 3:** Categories of features included in prognostic models across both reviews.

Study	Demographics	Medical history	Symptom severity	Stroke type	Imaging findings	Acute medical complications	Laboratory markers	Baseline function	Health factors
*Post-stroke cognitive impairment and dementia*									
Chander (2017)^52^	∗				∗				
Ding (2019)^53^	∗	∗			∗				
Gong (2019)^57^			∗	∗	∗				
Kandiah (2016)^54^	∗				∗				
Lin (2003)^55^	∗	∗	∗	∗				∗	
Munsch (2016)^56^	∗		∗		∗				
Salihovic (2018)^58^								∗	
Ashburner 2024 (A)^33^	∗	∗							∗
Ashburner 2024 (B)^33^	∗	∗							∗
Ashburner 2024 (C)^33^	∗	∗							∗
Molad 2019 (A)^43^	∗	∗			∗				∗
Molad 2019 (B)^43^					∗		∗		
Chu 2023^35^	∗	∗	∗						
Dharmasaroja 2022^36^	∗	∗		∗	∗				
Georgakis 2023 (A)^37^	∗	∗	∗		∗			∗	
Georgakis 2023 (B)^37^	∗	∗	∗		∗			∗	
Georgakis 2023 (C)^37^	∗	∗	∗		∗		∗	∗	∗
Gong 2021^38^	∗				∗		∗		
Huang 2022^40^	∗	∗	∗		∗			∗	
Lee 2023^41^	∗	∗	∗	∗	∗		∗	∗	∗
Ma 2022^42^	∗	∗					∗		
Pan 2023 (A)^44^	∗		∗		∗				
Pan 2023 (B)^44^	∗		∗		∗				
Wang 2024 (A)^45^	∗				∗				
Wang 2024 (B)^45^							∗		
Wang 2024 (C)^45^	∗				∗		∗		
Wang 2024 (D)^45^	∗				∗		∗		
Zhao 2024 (A)^47^	∗	∗	∗		∗				
Zhao 2024 (B)^47^	∗	∗	∗		∗		∗		
Zhao 2024 (C)^47^	∗	∗	∗		∗		∗		
Zhao 2024 (D)^47^	∗	∗	∗		∗		∗		
Zhao 2024 (E)^47^	∗	∗	∗		∗		∗		
Zhao 2024 (F)^47^	∗	∗	∗		∗		∗		
Aamodt 2021^32^	∗	∗	∗		∗				
Betrouni 2022^34^	∗				∗			∗	
Hasan 2024^39^	∗	∗							
Yuan 2021^46^	∗	∗			∗				
Zhu 2020^48^	∗	∗						∗	
*Post-stroke delirium*									
Kostalova (2012) (1)^59^	∗			∗	∗		∗		
Kotsalova (2012) (2)^59^	∗			∗	∗	∗			
Kotfis (2019)^60^	∗		∗				∗	∗	
Oldenbeuving (2014)^61^	∗		∗	∗		∗			
Guldolf 2021^49^	∗	∗	∗				∗	∗	
Haight 2020^50^	∗				∗	∗	∗	∗	
Klimiec-Moskal 2022^51^ (A)	∗		∗						
Klimiec-Moskal 2022^51^ (B)	∗		∗				∗		
Klimiec-Moskal 2022^51^ (C)		∗	∗	∗					∗
Klimiec-Moskal 2022^51^ (D)		∗	∗	∗			∗		∗

**Key:** Grey Background = Previous Review; White Background = Current Review.

The discriminative accuracy of the models ranged from poor (Cox regression; AUC of 0.58^43^) to excellent (Logistic regression; AUC 0.97^42^). Three models were externally validated in a separate population from the derivation cohort (C-statistic: 0.72 (0.68–0.77)–0.91 (no 95% CI reported)^33^^,^^39^^,^^46^ with one model undergoing temporal validation (AUC 0.81).^38^ From the seventeen studies, seven studies underwent assessment for calibration with all models showing good calibration.^35^, ^36^, ^37^, ^38^, ^39^, ^40^^,^^46^

### Prediction scores for post-stroke delirium

Similar to the previous review, the three new studies with models designed to predict post-stroke delirium between 72 h^51^ and 7 days^49^^,^^50^ did not exclude dementia or cognitive impairment at baseline. The number of features ranged from 2^51^ to 7.^49^ Across both reviews, all six studies^49^, ^50^, ^51^^,^^59^, ^60^, ^61^ used demographic features (such as age) with symptom severity (measured by the National Institutes of Health Stroke Scale) being used in 4 of the studies^49^^,^^51^^,^^60^^,^^61^ (Supplementary Table S2). Like models for PSCI and dementia, the laboratory markers were infection or inflammatory markers.^49^^,^^51^^,^^60^ Across both reviews there are 27 unique variables (demographics (n = 1), medical history (n = 5), symptom severity (n = 1), stroke type (n = 3), imaging (n = 2), acute medical complications (n = 6), laboratory markers (n = 7), Baseline Function (n = 2)). There were three studies capturing six new models for post-stroke delirium. All models were developed with logistic regression with moderate (AUC 0.77 (95% CI 0.71–0.81)^51^ to high levels (AUC 0.9 (no 95% CI reported))^50^ of discriminative accuracy. One of the models did perform temporal validation^50^ but none performed external validation. Two of the models from one study reported good calibration.^51^

### Meta-analysis of model performance

**Fig. 3:**
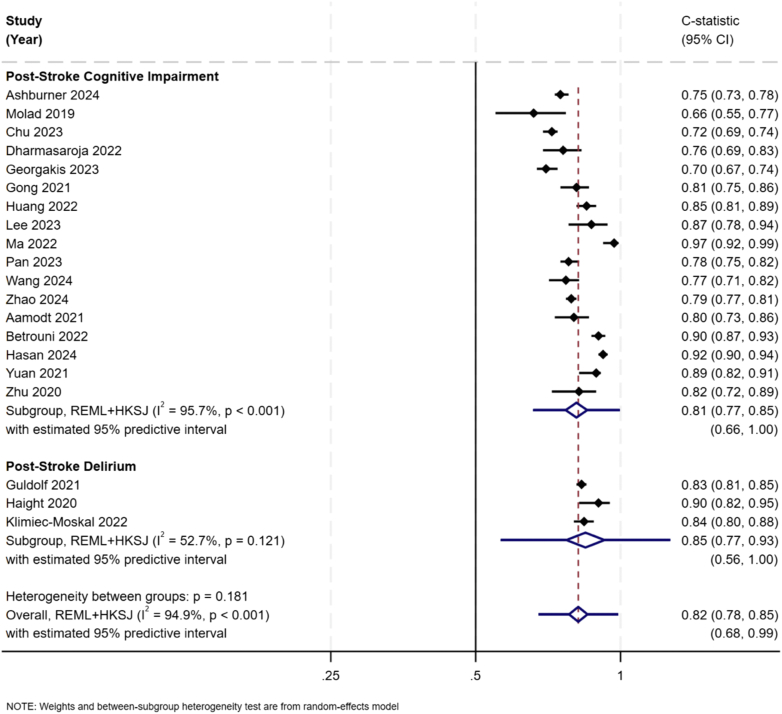
**Forest plot of discriminatory accuracy by outcome**.

Fig. 3 summarises the meta-analysis of model performance for post-stroke cognitive impairment (17 studies) and delirium (3 studies) outcomes. The median c-statistic (or equivalent AUROC) for cognitive impairment was 0.80 (IQR: 0.75, 0.97) and for delirium was 0.84 (IQR: 0.83, 0.90). The pooled c-statistic for post-stroke cognitive impairment and delirium were 0.81 (95% CI 0.77–0.85, *I*^*2*^ 95.7%) and 0.85 (95% CI 0.77–0.93, *I*^*2*^ 52.7%), respectively. For both outcomes, 95% prediction interval was wide indicating varied model performances are expected in a new study (cognitive impairment: 0.66–1.00); and delirium: 0.56–1.00). In a subgroup analysis for post-stroke cognitive impairment outcome (Supplementary Figure S1), pooled c-statistic was found to be lower when models fitted using regression techniques (0.79, 95% CI 0.74–0.84, *I*^*2*^ 94.5%) compared to machine learning methods (0.88, 95% CI 0.82–0.94, *I*^*2*^ 72.8%). Potential extent of publication bias for the post-stroke cognitive impairment outcome presented in the funnel plot (Supplementary Figure S2) that indicates variation across the funnel however small-study effect does not show any statistical significance (p = 0.331). Therefore, it should be interpreted with caution. In meta-regression analysis, bubble plots show potential heterogeneity in each moderating factor (i.e., age, sample size, number of observed events, and follow-up time) however, none of these factors reached statistical significance (Supplementary Table S3 and Supplementary Figure S3).

### Overall quality and certainty of the evidence

Seventeen studies^32^^,^^34^, ^35^, ^36^, ^37^, ^38^^,^^40^, ^41^, ^42^, ^43^, ^44^, ^45^, ^46^, ^47^, ^48^, ^49^, ^50^ had low risk of bias across all domains, while three studies^33^^,^^39^^,^^51^ had a high risk of bias due to outcome misclassification (Table 4). This was primarily from reliance on ICD-coded diagnoses rather than standardised cognitive screening tools such as Montreal Cognitive Assessment (MOCA) or Mini-Mental State examination (MMSE). At present there are currently no clearly superior cognitive screening tests and in particular MMSE is no worse than other screening tools for the diagnosis of multidomain impairment.^62^ Applicability concerns were also noted in these three studies, as outcome assessment methods may limit clinical generalisability.^33^^,^^39^^,^^51^ We performed GRADE assessment across prediction models for PSCI and delirium separately across both reviews. Overall, the certainly of the evidence is low across both PSCI and delirium models due to heterogeneity across the studies, the range of values for discrimination outcomes and also a lack of pre-registered protocols (Supplementary Table S3).

**Table 4:** Risk of bias assessment using the PROBAST tool.

	Risk of bias					Applicability			
	Participants	Predictors	Outcomes	Analysis	Overall	Participants	Predictors	Outcome	Overall
Aamodt 2021^32^	**+**	**+**	**+**	**+**	**+**	**+**	**+**	**+**	**+**
Ashburner 2024^33^	**+**	**+**	**-**	**+**	**-**	**+**	**+**	**-**	**-**
Betrouni 2022^34^	**+**	**+**	**+**	**+**	**+**	**+**	**+**	**+**	**+**
Chu 2023^35^	**+**	**+**	**+**	**+**	**+**	**+**	**+**	**+**	**+**
Dharmasaroja 2022^36^	**+**	**+**	**+**	**+**	**+**	**+**	**+**	**+**	**+**
Georgakis 2023^37^	**+**	**+**	**+**	**+**	**+**	**+**	**+**	**+**	**+**
Gong 2021^38^	**+**	**+**	**+**	**+**	**+**	**+**	**+**	**+**	**+**
Guldolf 2021^49^	**+**	**+**	**+**	**+**	**+**	**+**	**+**	**+**	**+**
Haight 2020^50^	**+**	**+**	**+**	**+**	**+**	**+**	**+**	**+**	**+**
Hasan 2024^39^	**+**	**+**	**-**	**+**	**-**	**+**	**+**	**-**	**-**
Huang 2022^40^	**+**	**+**	**+**	**+**	**+**	**+**	**+**	**+**	**+**
Klimiec-Moskal 2022^51^	**+**	**+**	**-**	**+**	**-**	**+**	**+**	**-**	**-**
Lee 2023^41^	**+**	**+**	**+**	**+**	**+**	**+**	**+**	**+**	**+**
Ma 2022^42^	**+**	**+**	**+**	**+**	**+**	**+**	**+**	**+**	**+**
Molad 2019^43^	**+**	**+**	**+**	**+**	**+**	**+**	**+**	**+**	**+**
Pan 2023^44^	**+**	**+**	**+**	**+**	**+**	**+**	**+**	**+**	**+**
Wang 2024^45^	**+**	**+**	**+**	**+**	**+**	**+**	**+**	**+**	**+**
Yuan 2021^46^	**+**	**+**	**+**	**+**	**+**	**+**	**+**	**+**	**+**
Zhao 2024^47^	**+**	**+**	**+**	**+**	**+**	**+**	**+**	**+**	**+**
Zhu 2020^48^	**+**	**+**	**+**	**+**	**+**	**+**	**+**	**+**	**+**

## Discussion

To our knowledge, this is the most comprehensive review describing models to predict PSCI, post-stroke dementia and post-stroke delirium. Pooled analysis showed that model discrimination was good with some evidence that machine learning methods are generally higher. However, some caution is needed when considering clinical utility of these models. There was significant heterogeneity between studies. Further, although there has been an increase in model development, these were generally in small samples and in the main from Asia. Further, few models were developed in line with best practice guidance.^63^ Accurate and timely identification of those most at risk enables that these individuals have the opportunity to modify their risk through multimodal interventions which have already been shown to be effective for those at-risk of dementia in the general population.^64^

When combining these results with the previous review,^20^ there are now a total of 38 models to predict PSCI and post-stroke dementia and 10 models for predicting post-stroke delirium. Only two models undertook full evaluation with discrimination, calibration and external validation in PSCI.^39^^,^^46^ Although discrimination and stability of the models were good (AUC >0.80), like other models, these development cohorts were generally relatively small with only 3 models with over a thousand participants in their development cohort (n: 677, range: 2234^39^-376^46^) when compared to risk modelling for other diseases. Another important aspect of model development is external validation and yet the validation cohorts were even smaller (n = 975^39^ and 227^46^). Finally, there were some considerations around the accessibility of models, In one study, they avoided the use of specialist imaging markers and instead used primary care datasets to develop a model for PSCI, which demonstrated moderate levels of discriminatory accuracy.^33^

Development of stroke specific risk models is needed so clinicians are able to identify those at-risk of cognitive decline post-stroke to ensure timely access to risk reduction strategies.^65^ Even though there are a significant number of models developed in whole populations for dementia prediction,^23^^,^^66^ they do not work well in stroke-survivors, which may be related to the risk factors that are included.^67^ Given the rapid increase in both interest and methodological development in dementia risk models for the general population, it was important to update the previous review to capture any new models developed specifically for stroke patients. Compared to the previous review, there has been a significant increase in the number of models which utilise machine learning techniques. Many of these models have displayed higher level of discriminative accuracy compared to traditional regression models as demonstrated in our pooled analysis. Machine learning has already been used to predict dementia in the general population with some evidence that these methods show better performance when these approaches are based on imaging data rather than clinical variables.^68^ Though machine learning models tend to produce good levels of discriminative accuracy, one criticism is the lack of clinical interpretability. A review identified 92 studies that applied interpretable methods to machine learning models but tended to focus on single open-source datasets.^69^ In this study there were attempts to try and make the models more interpretable. *Lee* et al. utilised the SHapley Additive exPlanations values of the best prediction model which was their Extreme Gradient Boosting (XGB) model.^41^ Traditional factors that did rank highly included for example discharge stroke severity and age. However, diabetes ranked much lower when compared to non-evidenced based scores such as the short geriatric score ranking higher even though diabetes has consistently been significantly associated with PSCI.^70^ Further, models tend to be developed with variables that are available within the dataset rather than focusing on evidence based known risk factors to build the models irrespective of what statistical methods are used. Some models, particularly the ones using machine learning, utilised many features (n = 30).^41^ This can lead to overfitting the model due to the volume of variables used. In model development it is therefore important to ensure best practice is followed and to take into account multiple parameters in model assessment rather than simply discrimination. This would include metrics such as calibration and decision curve analysis for example which are inconsistently reported. At present the evidence around models for delirium is much less advanced than models for PSCI and dementia both in terms of the types of models produced and the size of the dataset. Part of this may be due to the challenges around recognising and diagnosing delirium. Hypoactive stroke-survivors can often be confused as having post-stroke depression and fatigue.^17^ Further there may be issues in recognising delirium post-stroke despite how highly prevalent it is in the acute setting.^71^ Further work is needed to develop risk prediction models in this area to ensure early recognition is possible to ensure appropriate intervention is in place.

Known risk factors for PSCI and dementia often go beyond traditional features e.g. age and stroke severity. A recent systematic review concluded that baseline cognitive impairment showed the strongest association with both PSCI and post-stroke dementia.^70^ As we excluded models where baseline cognitive impairment and dementia were included, it is not surprising that very few (n = 3) of the models included in this study for PSCI and dementia contained this feature. Two of the models to predict post-stroke delirium did include a feature associated with baseline cognitive deficit.^49^^,^^50^ Other factors that increase risk of PSCI and dementia in the context of stroke include diabetes, atrial fibrillation and the presence of moderate or severe white matter hyperintensities.^49^^,^^50^ Across both reviews diabetes (n = 8)^35^^,^^37^^,^^39^^,^^41^, ^42^, ^43^^,^^46^^,^^53^ was the most common medically related risk factor to be included. Atrial fibrillation was only included in three models^37^^,^^41^^,^^43^ and white matter hyperintensities (or Fazeka score) in nine studies.^36^, ^37^, ^38^^,^^41^^,^^43^^,^^45^^,^^47^^,^^52^^,^^54^ Lower years of education and previous stroke are also known to increase risk for PSCI^70^ and again are well represented across the models (education n = 16^32^^,^^35^, ^36^, ^37^, ^38^^,^^40^, ^41^, ^42^^,^^44^, ^45^, ^46^, ^47^, ^48^^,^^52^^,^^53^^,^^54^ and previous stroke n = 7^32^^,^^36^^,^^37^^,^^41^^,^^46^^,^^47^^,^^55^). Ideally, the models themselves would contain modifiable risk factors but there needs to be a balance between discriminative ability and risk factor accessibility. This may depend on the purpose of the tool i.e., whether it is purely for identification only. Future studies should consider a component meta-analysis of these multicomponent risk prediction scores to assess the importance of risk factors across models.

One criticism of risk prediction models for PSCI including in the context of stroke is knowing what can be done to lower one's risk.^72^ Including modifiable risk factors such as diabetes, atrial fibrillation and preventing recurrent stroke, all factors known to increase risk of PSCI, should be at the forefront of any interventions as well as being used in model development. Although many high value risk factors (such as baseline cognition and education) may not be modifiable, strong predictive models can help ensure early identification, psychoeducation, monitoring and management of patients, expanding upon the existing narrow focus on medication management and secondary prevention in long term stroke care.^73^ Primary care is often responsible for managing these conditions. Use of features that can be accessed and analysed by primary care services would provide the greatest potential for intervention. Only one study utilised primary care clinical records to develop a model for PSCI or dementia with moderate levels of accuracy (Full model: C-statistic 0.75 (95% CI: 0.73–0.78).^33^ However, besides age, the remaining features are not known to be clearly associated with PSCI and post-stroke dementia. Future studies could look to utilise evidence-based risk factors for PSCI and dementia to develop models in large volume primary care datasets.

Development of all risk models has tended to be in relatively small cohorts. Although cohort sizes have increased since the first review, across both reviews, most studies had less than a thousand stroke-survivors in their development cohort. When developing a prediction model, the sample size depends on the disease prevalence in the study population, candidate predictor parameters, and desired percentage of variation in outcome values explained by the model (commonly reported by R-squared). The models included in this review often lacked formal methodological approaches to determine sample size criteria and did not adhere to good practice.^74^^,^^75^ This is particularly important when considering how many variables should be included in the overall prediction model. Events per variable (EPV) have previously been used to address this but simulation studies have shown that EPV rules for binary logistic regression is weak^76^ and large sample sizes are needed when using machine learning methods.^77^ Future studies should consider parameters such as the number of predictors, total sample size and events fraction as criteria in the development of their model.^76^ To address the issue around small cohorts, there have been some efforts to try and harmonise stroke cohorts. For example, one study harmonised three stroke cohorts as part of the STROKOG consortium.^34^ A similar approach has been used to externally validate simple dementia risk models previously.^67^ However, given that these are small cohorts model development will often be restricted by common features across all cohorts. The model that harmonised these three cohorts had only 327 participants. Large datasets, in the form of electronic healthcare records are likely to be needed to take the next step in the field of PSCI and dementia risk prediction alongside explainable modelling techniques to find non-linear relationships between features and unravel the complexities between these relationships. This will be particularly important when we begin to consider non-traditional risk factors as there is evidence to suggest that higher order factors such as emotional distress and subjective health are more important than defined clinical factors when evaluated together.^78^ This is likely because such higher-order factors reflect the complex interactions between functional, social, mental and biological aspects of the individual.^78^ Another aspect that needs considering in model development is how the primary study addressed missing data. We did not formally assess how included papers treated missing data in their models. However, we noted that this aspect was poorly reported and where an approach was described there was substantial heterogeneity in the method used. In studies of populations with stroke and cognitive issues, the missing data is likely to relate to the exposure and the outcome, and so missing data are an important threat to the validity of the results. Finally, methodologically few of the models developed actually followed best practice in terms of both model development^63^ and validation.^79^ Although all studies included had measures of discrimination, few had other robust measures of model assessment including calibration and decision curve analysis^80^ which may limit the current models’ clinical utility.

The strengths of this study include the inclusive search strategy to capture all available models, regardless of language and alignment with our previous review criteria, to enable model comparison from both reviews. Studies that included pre-morbid cognitive impairment and those that did not specify whether the population included those with baseline cognitive impairment or dementia were excluded to ensure homogeneity in our final selection of studies. Although there will be stroke patients who have pre-existing cognitive impairment prior to their stroke, the models presented in this and the previous review reflect a proportion of the stroke population where there is no cognitive impairment at baseline. We do appreciate that the findings here may not be directly applicable to those with pre-existing cognitive impairment at baseline and future studies should look to synthesise these findings. The previous review did include a study that predicted “no cognitive impairment”. We have included this study in this review given that it met the original inclusion criteria, but we did not include studies that predicted non-cognitive impaired outcomes or recovery. Furthermore, we kept the delirium models separate from the PSCI and post-stroke dementia studies as the delirium models did not exclude cognitive impairment at baseline, in line with the previous review. We also performed an updated search (Supplementary Table S4) which further highlights the rapid development of such models but in general reflects the ongoing methodological limitations encountered by the models included in the formal analysis. We do recognise some limitations. The findings were limited by geographical imbalance and related differences in population demographics, lack of external validation, and methodological heterogeneity between included studies. Further, our search only included published studies and grey literature was not included. While dementia risk models for the general population are predominantly developed in high-income settings, most of the models developed for cognitive syndromes in our review originated from upper-middle-income China, with additional contributions from other Asian countries, such as Thailand and South Korea. Few models were developed in high-income settings such as the US, UK and Germany. This could potentially restrict the generalisability of our findings outside of Asia, particularly as the risk profile for certain comorbidities associated with post-stroke cognitive decline may differ between Asian and non-Asian ethnicities. Methodologically, very few studies were externally validated in populations separate to the derivation cohort which limits our understanding as to how transportable these models are in other settings and particularly how accurate they are in other non-Asian populations. Further, although we did exclude those with pre-stroke cognitive impairment and dementia so that we could compare across both reviews, we do recognise the frequency of pre-stroke cognitive impairment. It may also be helpful to find ways to determine which patients with pre-stroke cognitive impairment go on to develop dementia. This is because pre-stroke cognitive impairment is both a major risk factor for post-stroke cognitive impairment and is frequently found prior to the index stroke event. However, pre-stroke cognitive assessment is difficult due to imprecise assessment tools and screening is unfortunately rarely performed in clinical practice. Although it is likely that there may be those with undetected pre-stroke cognitive impairment in clinical settings, the intention of this study was to synthesise existing clinical prediction scores where symptomatic dementia/cognitive impairment was already present at baseline. Finally, we could not account for all methodological difference between all the included studies, specifically in how PSCI and dementia were diagnosed across studies and researcher settings.

An accurate, cost-effective and clinical useable model is key if we are to identify those stroke patients at the greatest risk of cognitive decline. This will not only have an impact on risk reduction strategies being developed and implemented, but this could also potentially reduce the overall numbers of stroke patients with cognitive decline which has significant healthcare, economic and societal cost implications. Despite the significant increase in model development, overall certainty of evidence is low and external validation to assess model transportability is unfortunately still lacking. This is particularly important for models where machine learning approaches are used. There is a risk of overfitting if external validation is not a routine part of model development. Further, few models followed best practice guidance for model development^63^ and validation^79^ which is recommended to ensure clinical utility. The predominance of models being developed in Asia without external validation in other populations limits any current recommendations of any model for clinical usage. Future research needs to utilise large datasets which capture a diverse population such as primary care records and focus on all aspects of model development from discrimination and calibration to full external validation. Upon model development, it is essential that cost effectiveness is assessed. Model developed should also involve key stakeholders to further test and evaluate implementing models into clinical practice.

## Contributors

EYHT and TQ conceptualised the idea for this manuscript. The information specialist (LE) conducted the searches based on the agreed search terms. RDI, SS, FM, EJ screened at title and abstract stage and RDI, SS, FM, EJ, JD, CB, LG, JB screened at full text review. EYHT and JB were responsible for data extraction. EYHT and JB accessed and verified the data. JB assessed risk of bias and TQ performed GRADE assessment. RB performed the meta-analysis. EYHT prepared the manuscript. All authors reviewed and agreed on the contents of the manuscript.

## Data sharing statement

All data for this review is contained within the manuscript.

## Declaration of interests

RB declares funding to their institution from NIHR Health and Social Care Delivery Research (HSDR) Programme and NIHR School for Primary Care Research (SPCR); participation on a Data Safety Monitoring Board or Advisory Board (Chair DMC for Predict & Prevent AECOPD Trial); and a leadership or fiduciary role in other board, society, committee or advocacy group, paid or unpaid (College of Expert, Versus Arthritis Selection Committee Member, NIHR Pre-doctoral Fellowship). TQ declares participation on a Data Safety Monitoring Board or Advisory Board (Novo Nordisk–Chair of DSMB for dementia trials portfolio; no personal payment); and a leadership or fiduciary role in other board, society, committee or advocacy group, paid or unpaid (Chair of European Stroke Organisation Guideline Group, includes guidelines on stroke cognitive issues). ND declares funding to their institution from the Stroke Association and Brain Stimulation (SE); consulting fees from Brain Stimulation (SE) and Arega NV; payment or honoraria for lectures, presentations, speakers bureaus, manuscript writing or educational events (University of Glasgow and University of Edinburgh); support for attending meetings and/or travel (ANZSOC and UK Stroke Forum); Leadership or fiduciary role in other board, society, committee or advocacy group, paid or unpaid (Elsevier—remunerated section editor). CP declares research awards from NIHR RfPB and PGfAR. All other authors declare no competing interests.
