# Supplementary material for: Clinical prediction rules for cognitive outcomes post-stroke: an updated systematic review and meta-analysis
Source: eClinicalMedicine. 2025 Nov 25;90:103664. doi: 10.1016/j.eclinm.2025.103664 (PMC12702050; doi:10.1016/j.eclinm.2025.103664)
Supplement: Supplementary Figure and Tables [file mmc1.docx]

**Supplementary Material**

**Search Strategy**

We utilised the same search terms as per the original study

Medline (via OVID) search strategy:

1. cerebrovascular disorders/ OR exp basal ganglia cerebrovascular disease/ OR exp brain ischemia/ OR exp intracranial arterial diseases/ OR exp "intracranial embolism and thrombosis"/ OR exp intracranial hemorrhages/ OR stroke/ OR exp brain infarction/ OR vasospasm, intracranial/
2. (stroke OR post?stroke OR cerebrovasc$ OR brain vasc$ OR cerebral vasc$ OR cva$ OR apoplex$ OR SAH).ti,ab.
3. ( (brain$ OR cerebr$ OR cerebell$ OR intracran$ OR intracerebral) adj5 (isch?emi$ OR infarct$ OR thrombo$ OR emboli$ OR occlus$)).ti,ab.
4. ( (brain$ OR cerebr$ OR cerebell$ OR intracerebral OR intracranial OR subarachnoid) adj5 (h?emorrhage$ OR h?ematoma$ OR bleed$)).ti,ab.
5. ( (transi$ adj3 isch?em$ adj3 attack$) OR TIA$1).ti,ab.
6. 1OR2OR3OR4OR5
7. ( (validat$ OR predict$ OR prognos$ OR rule$) adj3 (outcome$ OR risk$ OR

model$)).ti,ab.

1. (prognos$ AND (method$ OR history OR variable$ OR criteria OR scor$ OR

characteristic$ OR finding$ OR factor$ OR model$)).ti,ab.

1

1. ( (history OR variable$ OR criteria OR scor$ OR characteristic$ OR finding$ OR factor$) adj3 (predict$ OR model$ OR decision$ OR identif$ OR prognos$)).ti,ab.
2. (decision$ adj3 (model$ OR clinical$)).ti,ab.
3. (stratification OR discriminat$ OR calibration).ti,ab.
4. ROC curve/
5. (c-statistic OR c statistic OR area under the curve OR AUC).ti,ab.
6. (indices OR algorithm OR multivariable).ti,ab.
7. 7 OR 8 OR 9 OR 10 OR 11 OR 12 OR 13 OR 14
8. exp dementia/
9. delirium/
10. delirium, dementia, amnestic, cognitive disorders/
11. exp cognition disorders/
12. exp cognition/
13. memory/
14. dement$.ti,ab.
15. (Alzheimer$ OR AD).ti,ab.
16. deliri$.ti,ab.
17. ( (cognit$ OR memory OR mental OR brain) adj3 (func$ OR perform$ OR abilit$ OR

declin$ OR reduc$ OR impair$ OR disorder$ OR fail$ OR los$ OR deficit$ OR stop$ OR progress$ OR improve$)).ti,ab.

Embase (via OVID) search strategy:

1. cerebrovascular disease/ OR exp basal ganglion hemorrhage/ OR exp brain hematoma/ OR exp brain hemorrhage/ OR exp brain infarction/ OR exp brain ischemia/ OR cerebral artery disease/ OR exp cerebrovascular accident/ OR exp occlusive cerebrovascular disease/ OR vertebrobasilar insufficiency/ OR stroke/ OR stroke patient/ OR stroke unit/

26. mental perform$.ti,ab.
27. (memory adj3 (complain$ or declin$ or function$)).ti,ab.
28. 16 OR 17 OR 18 OR 19 OR 20 OR 21 OR 22 OR 23 OR 24 OR 25 OR 26 OR 27 29. 6 AND 15 AND 28

2

1. (stroke OR post?stroke OR cerebrovasc$ OR brain vasc$ OR cerebral vasc$ OR cva$ OR apoplex$ OR SAH).ti,ab.
2. ( (brain$ OR cerebr$ OR cerebell$ OR intracran$ OR intracerebral) adj5 (isch?emi$ OR infarct$ OR thrombo$ OR emboli$ OR occlus$)).ti,ab.
3. ( (brain$ OR cerebr$ OR cerebell$ OR intracerebral OR intracranial OR subarachnoid) adj5 (h?emorrhage$ OR h?ematoma$ OR bleed$)).ti,ab.
4. ( (transi$ adj3 isch?em$ adj3 attack$) OR TIA$1).ti,ab.
5. 1OR2OR3OR4OR5
6. ( (validat$ OR predict$ OR prognos$ OR rule$) adj3 (outcome$ OR risk$ OR

model$)).ti,ab.

1. (prognos$ AND (method$ OR history OR variable$ OR criteria OR scor$ OR

characteristic$ OR finding$ OR factor$ OR model$)).ti,ab.

1. ( (history OR variable$ OR criteria OR scor$ OR characteristic$ OR finding$ OR

factor$) adj3 (predict$ OR model$ OR decision$ OR identif$ OR prognos$)).ti,ab.

1. (decision$ adj3 (model$ OR clinical$)).ti,ab.
2. (stratification OR discriminat$ OR calibration).ti,ab.
3. receiver operating characteristic/
4. (c-statistic OR c statistic OR area under the curve OR AUC).ti,ab.
5. (indices OR algorithm OR multivariable).ti,ab.
6. 7 OR 8 OR 9 OR 10 OR 11 OR 12 OR 13 OR 14
7. exp dementia/
8. delirium/
9. exp cognitive defect/
10. exp cognition/
11. memory/
12. dement$.ti,ab.
13. (Alzheimer$ OR AD).ti,ab.
14. deliri$.ti,ab.

24. ( (cognit$ OR memory OR mental OR brain) adj3 (func$ OR perform$ OR abilit$ OR

declin$ OR reduc$ OR impair$ OR disorder$ OR fail$ OR los$ OR deficit$ OR stop$ OR

progress$ OR improve$)).ti,ab.

25. mental perform$.ti,ab.

3

26. (memory adj3 (complain$ or declin$ or function$)).ti,ab.
27. 16 OR 17 OR 18 OR 19 OR 20 OR 21 OR 22 OR 23 OR 24 OR 25 OR 26 28. 6 AND 15 AND 27

PsycINFO (via EBSCO) search strategy:

S1 DE "Cerebrovascular Disorders" OR DE "Cerebral Arteriosclerosis" OR DE "Cerebral Hemorrhage" OR DE "Cerebral Ischemia" OR DE "Cerebrovascular Accidents" OR DE "Subarachnoid Hemorrhage"

S2 TI (stroke OR post#stroke OR cerebrovasc* OR "brain vasc*" OR "cerebral vasc*" OR cva* OR apoplexy OR SAH) OR AB (stroke OR post#stroke OR cerebrovasc* OR "brain vasc*" OR "cerebral vasc*" OR cva* OR apoplexy OR SAH)

S3 TI ( (brain* OR cerebr* OR cerebell* OR intracran* OR intracerebral) N5 (isch#emi$ OR infarct* OR thrombo* OR emboli* OR occlus*)) OR AB ( (brain* OR cerebr* OR cerebell* OR intracran* OR intracerebral) N5 (isch#emi$ OR infarct* OR thrombo* OR emboli* OR occlus*))

S4 TI ( (brain* OR cerebr* OR cerebell* OR intracerebral OR intracranial OR subarachnoid) N5 (h#emorrhage* OR h#ematoma* OR bleed*)) OR AB ( (brain* OR cerebr* OR cerebell* OR intracerebral OR intracranial OR subarachnoid) N5 (h#emorrhage* OR h#ematoma* OR bleed*))

S5 TI ( (transi* N3 isch#em* N3 attack*) OR TIA) OR AB ( (transi* N3 isch#em* N3 attack*) OR TIA)

S6S1ORS2ORS3ORS4ORS5

S7 TI ( (validat* OR predict* OR prognos* OR rule*) N3 (outcome* OR risk* OR model*)) OR AB ( (validat* OR predict* OR prognos* OR rule*) N3 (outcome* OR risk* OR model*))

S8 TI (prognos* AND (method* OR history OR variable* OR criteria OR scor* OR characteristic* OR finding* OR factor* OR model*)) OR AB (prognos* AND (method* OR history OR variable* OR criteria OR scor* OR characteristic* OR finding* OR factor* OR model*))

4

S9 TI ( (history OR variable* OR criteria OR scor* OR characteristic* OR finding* OR factor*) N3 (predict* OR model* OR decision* OR identif* OR prognos*)) OR AB ( (history OR variable* OR criteria OR scor* OR characteristic* OR finding* OR factor*) N3 (predict* OR model* OR decision* OR identif* OR prognos*))

S10 TI (decision* N3 (model* OR clinical*)) OR AB (decision* N3 (model* OR clinical*))

S11 TI (stratification OR discriminat* OR calibration) OR AB (stratification OR discriminat* OR calibration)

S12 TI ("c-statistic" OR "c statistic" OR "area under the curve" OR AUC) OR AB ("c- statistic" OR "c statistic" OR "area under the curve" OR AUC)

S13 TI (indices OR algorithm OR multivariable) OR AB (indices OR algorithm OR multivariable)

S14S7ORS8ORS9ORS10ORS11ORS12ORS13

S15 DE "Dementia" OR DE "Presenile Dementia" OR DE "Pseudodementia" OR DE "Semantic Dementia" OR DE "Senile Dementia" OR DE "Vascular Dementia"

S16 DE "Neurocognitive Disorders" OR DE "Delirium" OR DE "Memory Disorders" OR DE "Cognitive Impairment"

S17 DE "Memory" OR DE "Memory Decay"
S18 DE "Cognition"
S19 TI dement* OR AB dement*
S20 TI (alzheimer* OR AD) OR AB (alzheimer* OR AD) S21 TI deliri* OR AB deliri*

S22 TI ( ( (cognit* OR memory OR mental OR brain) N3 (func* OR perform* OR ability* OR declin* OR reduc* OR impair* OR disorder* OR fail* OR los* OR deficit* OR stop* OR progress* OR improve*))) OR AB ( ( (cognit* OR memory OR mental OR brain) N3 (func* OR perform* OR ability* OR declin* OR reduc* OR impair* OR disorder* OR fail* OR los* OR deficit* OR stop* OR progress* OR improve*)))

S23 TI "mental perform*" OR AB "mental perform*"

5

S24 TI ( (memory N3 (complain* or declin* or function*))) OR AB ( (memory N3 (complain* or declin* or function*)))

S25S15ORS16ORS17ORS18ORS19ORS20ORS21ORS22ORS23OR24

S26 S6 AND S14 AND S25

CINAHL (via EBSCO) search strategy:

S1 (MH "Cerebrovascular Disorders") OR (MH "Basal Ganglia Cerebrovascular Disease+") OR (MH "Cerebral Ischemia+") OR (MH "Intracranial Arterial Diseases+") OR (MH “Intracranial Embolism and Thrombosis+”) OR (MH “Intracranial Hemorrhage+") OR (MH “Stroke+) OR (MH “Cerebral Vasospasm”)

S2 TI (stroke OR post#stroke OR cerebrovasc* OR "brain vasc*" OR "cerebral vasc*" OR cva* OR apoplexy OR SAH) OR AB (stroke OR post#stroke OR cerebrovasc* OR "brain vasc*" OR "cerebral vasc*" OR cva* OR apoplexy OR SAH)

S3 TI ( (brain* OR cerebr* OR cerebell* OR intracran* OR intracerebral) N5 (isch#emi$ OR infarct* OR thrombo* OR emboli* OR occlus*)) OR AB ( (brain* OR cerebr* OR cerebell* OR intracran* OR intracerebral) N5 (isch#emi$ OR infarct* OR thrombo* OR emboli* OR occlus*))

S4 TI ( (brain* OR cerebr* OR cerebell* OR intracerebral OR intracranial OR subarachnoid) N5 (h#emorrhage* OR h#ematoma* OR bleed*)) OR AB ( (brain* OR cerebr* OR cerebell* OR intracerebral OR intracranial OR subarachnoid) N5 (h#emorrhage* OR h#ematoma* OR bleed*))

S5 TI ( (transi* N3 isch#em* N3 attack*) OR TIA) OR AB ( (transi* N3 isch#em* N3 attack*) OR TIA)

S6S1ORS2ORS3ORS4ORS5

S7 TI ( (validat* OR predict* OR prognos* OR rule*) N3 (outcome* OR risk* OR model*)) OR AB ( (validat* OR predict* OR prognos* OR rule*) N3 (outcome* OR risk* OR model*))

S8 TI (prognos* AND (method* OR history OR variable* OR criteria OR scor* OR characteristic* OR finding* OR factor* OR model*)) OR AB (prognos* AND (method*

6

OR history OR variable* OR criteria OR scor* OR characteristic* OR finding* OR factor* OR model*))

S9 TI ( (history OR variable* OR criteria OR scor* OR characteristic* OR finding* OR factor*) N3 (predict* OR model* OR decision* OR identif* OR prognos*)) OR AB ( (history OR variable* OR criteria OR scor* OR characteristic* OR finding* OR factor*) N3 (predict* OR model* OR decision* OR identif* OR prognos*))

S10 TI (decision* N3 (model* OR clinical*)) OR AB (decision* N3 (model* OR clinical*))

S11 TI (stratification OR discriminat* OR calibration) OR AB (stratification OR discriminat* OR calibration)

S12 (MH “ROC Curve”)

S13 TI ( ("c-statistic" OR "c statistic" OR "area under the curve" OR AUC)) OR AB ( ("c- statistic" OR "c statistic" OR "area under the curve" OR AUC))

S14 TI (indices OR algorithm OR multivariable) OR AB (indices OR algorithm OR multivariable)

S15S7ORS8ORS9ORS10ORS11ORS12ORS13ORS14
S16 (MH "Dementia+")
S17 (MH "Delirium")
S18 (MH “Delirium, Dementia, Amnestic, Cognitive Disorders”) S19 (MH “Cognition Disorders+”)

S20 (MH “Cognition”)
S21 (MH “Memory”) OR (MH “Memory Disorders”) S22 TI dement* OR AB dement*
S23 TI (alzheimer* OR AD) OR AB (alzheimer* OR AD) S24 TI deliri* OR AB deliri*

S25 TI ( ( (cognit* OR memory OR mental OR brain) N3 (func* OR perform* OR ability* OR declin* OR reduc* OR impair* OR disorder* OR fail* OR los* OR deficit* OR stop* OR progress* OR improve*))) OR AB ( ( (cognit* OR memory OR mental OR brain) N3

7

(func* OR perform* OR ability* OR declin* OR reduc* OR impair* OR disorder* OR fail* OR los* OR deficit* OR stop* OR progress* OR improve*)))

S26 TI "mental perform*" OR AB "mental perform*"

S27 TI ( (memory N3 (complain* or declin* or function*))) OR AB ( (memory N3 (complain* or declin* or function*)))

S28S16ORS17ORS18ORS19ORS20ORS21ORS22ORS23ORS24ORS25ORS26OR

S27

S29 S6 AND S15 AND S28

**Supplementary Table 1: Frequency of Features Reported in Studies Across the Original(20) and Current Review for PSCI and Dementia**

| **Feature** | **Frequency Reported in Studies** |
| --- | --- |
| **Demographics (n=5)** | |
| Age(33-45, 52-56) | 18 |
| Education(22, 32, 35-38, 40-42, 44, 46-48, 52-54) | 16 |
| Gender(35, 37-39, 41, 42, 44, 47) | 8 |
| Occupation(55) | 1 |
| Insurance(33) | 1 |
| **Medical History (n=31)** | |
| Diabetes or diabetic drugs(35, 37, 39, 41-43, 46, 53) | 8 |
| Previous stroke or TIA (including infarction or haemorrhage)(32, 36, 37, 41, 42, 46, 47, 55) | 8 |
| History of hypertension, systolic blood pressure or antihypertensive medication(37, 41, 43, 47) | 4 |
| History of cardiovascular, coronary/ischaemic heart disease (including disease of the circulatory system)(39-41, 43) | 4 |
| Atrial Fibrillation(37, 41, 43) | 3 |
| Body Mass Index(37, 41, 48) | 3 |
| Depression or Antidepressants(33, 39, 41) | 3 |
| Mobility problems(33) | 1 |
| History of falls(33) | 1 |
| Delirium(33) | 1 |
| Peripheral arterial disease(33) | 1 |
| Parkinson’s Disease(33) | 1 |
| Chronic kidney disease (severe)(33) | 1 |
| Abnormal weight loss or anorexia(33) | 1 |
| History of hyperlipidaemia(41) | 1 |
| Antiplatelets(32) | 1 |
| Disease of the nervous system(39) | 1 |
| Disease of the respiratory system(39) | 1 |
| Antihaemorrhagics(39) | 1 |
| Antithrombotics(39) | 1 |
| Drugs related to acidity(39) | 1 |
| Drugs related to functional gastrointestinal disorders(39) | 1 |
| Hypnotic(39) | 1 |
| Systemic use of antibacterials(39) | 1 |
| NSAID(39) | 1 |
| Stomatological preparations(39) | 1 |
| Ophthalmologicals(39) | 1 |
| Drugs for constipation(39) | 1 |
| Analgesics(39) | 1 |
| Cough/cold preparations(39) | 1 |
| Poorly ill-defined conditions(39) | 1 |
| **Symptom Severity (n=2)** | |
| National Institutes of Health Stroke Scale score(32, 35, 37, 40, 41, 44, 47, 55, 56) | 9 |
| Glasgow Coma Score(57) | 1 |
| **Stroke Type (n=4)** | |
| Intraventricular haemorrhage(57) | 1 |
| Vascular Territory Infarction(55) | 1 |
| Stroke subtype(36) | 1 |
| TOAST classification(41) | 1 |
| **Imaging (n=34)** | |
| Deep and Periventricular White Matter Hyperintensities/Fazeka’s scale(36-38, 41, 43, 45, 47, 52, 54) | 9 |
| Chronic Lacunes/Lacune count(37, 40, 43, 52, 54) | 5 |
| Stroke or Infarct or Lesion volume(32, 41, 44, 56) | 4 |
| Acute non-lacunar infarct(52-54) | 3 |
| Global Cortical Atrophy(52, 54) | 2 |
| Intracranial stenosis/Number of intracranial atherosclerotic stenosis(38, 54) | 2 |
| Cerebral microbleed counts (including any chronic microbleeds)(37, 41) | 2 |
| Cortical infarcts/lesions(38, 41) | 2 |
| Medical temporal atrophy score/total mesial temporal atrophy(40, 41) | 2 |
| Periventricular Hyperintensities(53) | 1 |
| Bleeding Volume(57) | 1 |
| Stroke location expressed as number of eloquent voxels from voxel-based lesion-symptom mapping maps(56) | 1 |
| Normalized stroke lesion volume (stroke lesion volume/total intracranial volume)(37) | 1 |
| Global SVD Score (lacunes, white matter hyperintensities, cerebral microbleeds and enlarged perivascular spaces)(37) | 1 |
| Grade of perivascular spaces(37) | 1 |
| Hippocampal volume(43) | 1 |
| Number of chronic lacunar infarcts(40) | 1 |
| Multiple lesions(41) | 1 |
| Left sided lesions(41) | 1 |
| Presence of subcortical lesion(41) | 1 |
| Presence of infratentorial lesion(41) | 1 |
| Presence of strategic lesion(41) | 1 |
| Location impact score(44) | 1 |
| Disconnection score(44) | 1 |
| Occipital th. (left)(32) | 1 |
| Temporal th. (left)(32) | 1 |
| Cingulate (right)(32) | 1 |
| Texture features kurtosis (entorhinal cortex)(34) | 1 |
| IDM (entorhinal cortex)(34) | 1 |
| Kurtosis (hippocampus)(34) | 1 |
| Entropy (hippocampus)(34) | 1 |
| Left frontal NAA/Cr(46) | 1 |
| Left thalamus NAA/Cr(46) | 1 |
| Left hippocampus NAA/Cr(46) | 1 |
| **Laboratory Markers (n=13)** | |
| Circulating low-density lipoprotein cholesterol [LDL-C] levels/Total Cholesterol(37, 41) | 2 |
| Serum albumin(42, 45) | 2 |
| Lymphocyte count/percentages(45, 47) | 2 |
| Systemic inflammatory response index (absolute counts of neutrophils, monocytes, and lymphocytes)(35) | 1 |
| Prognostic nutritional index (albumin and lymphocyte count)(45) | 1 |
| Systemic immune inflammation index (neutrophil, lymphocyte, and platelet counts)(47) | 1 |
| HbA1c(38) | 1 |
| APOE4 status(43) | 1 |
| Haemoglobin(41) | 1 |
| Creatinine(41) | 1 |
| Fasting blood sugar level(41) | 1 |
| Neutrophil percentages/values(47) | 1 |
| Neutrophil-to-lymphocyte ratio(47) | 1 |
| **Baseline Function (n=9)** | |
| MoCA(34, 37, 48) | 3 |
| Pre-stroke modified Rankin scale(37, 41) | 2 |
| MMSE score(48, 55) | 2 |
| Functional Independence Measure motor score(55) | 1 |
| Complex figure test score(81) | 1 |
| Narrative memory score(81) | 1 |
| Numerical memory score(81) | 1 |
| Pre-stroke cognitive function(40) | 1 |
| Short form geriatric depression scale(41) | 1 |
| **Health Factors (n=3)** | |
| Smoking status(37, 41, 43) | 3 |
| Discharge from the hospital to a facility(33) | 1 |
| Alcohol Consumption(37) | 1 |

**Supplementary Table 2: Frequency of Features Reported in Studies Across the Original(7) and this Current Review for Post-Stroke Delirium**

| **Feature** | **Frequency Reported in Studies** |
| --- | --- |
| **Demographics (n=1)** | |
| Age(49-51, 58-60) | 6 |
| **Medical History (n=5)** | |
| History of previous stroke(49) | 1 |
| Premorbid cognitive dysfunction(49) | 1 |
| Hearing problems(49) | 1 |
| Atrial Fibrillation(51) | 1 |
| Diabetes(51) | 1 |
| **Symptom Severity (n=1)** | |
| NIHSS score(49, 51, 59, 60) | 4 |
| **Stroke Type (n=3)** | |
| Intracerebral haemorrhage(58) | 1 |
| Stroke subtype(60) | 1 |
| Haemorrhagic stroke(51) | 1 |
| **Imaging (n=2)** | |
| Lesion volume(58) | 1 |
| Intraventricular haemorrhage(50) | 1 |
| **Acute Medical Complications (n=6)** | |
| Hemianopia(59) | 1 |
| Aphasia(59) | 1 |
| Infection(60) | 1 |
| Intubation(50) | 1 |
| Stroke with either cognitive deficit, neglect, or aphasia(50) | 1 |
| SOFA-Max(58) | 1 |
| **Laboratory Markers (n=7)** | |
| C-reactive protein(51, 59) | 2 |
| Gamma-glutamyl transferase(58) | 1 |
| Bilirubin(58) | 1 |
| Neutrophil to lymphocyte ratio(49) | 1 |
| Leukocytes(59) | 1 |
| Neutrophil-lymphocyte ratio(49) | 1 |
| Presence of acute kidney injury(50) | 1 |
| **Baseline Function (n=2)** | |
| Premorbid modified Rankin score(49) | 1 |
| Pre-stroke dependency(51) | 1 |

**Supplementary Figure 1. Forest Plot of Discriminatory Accuracy by Type Modelling Used**

**
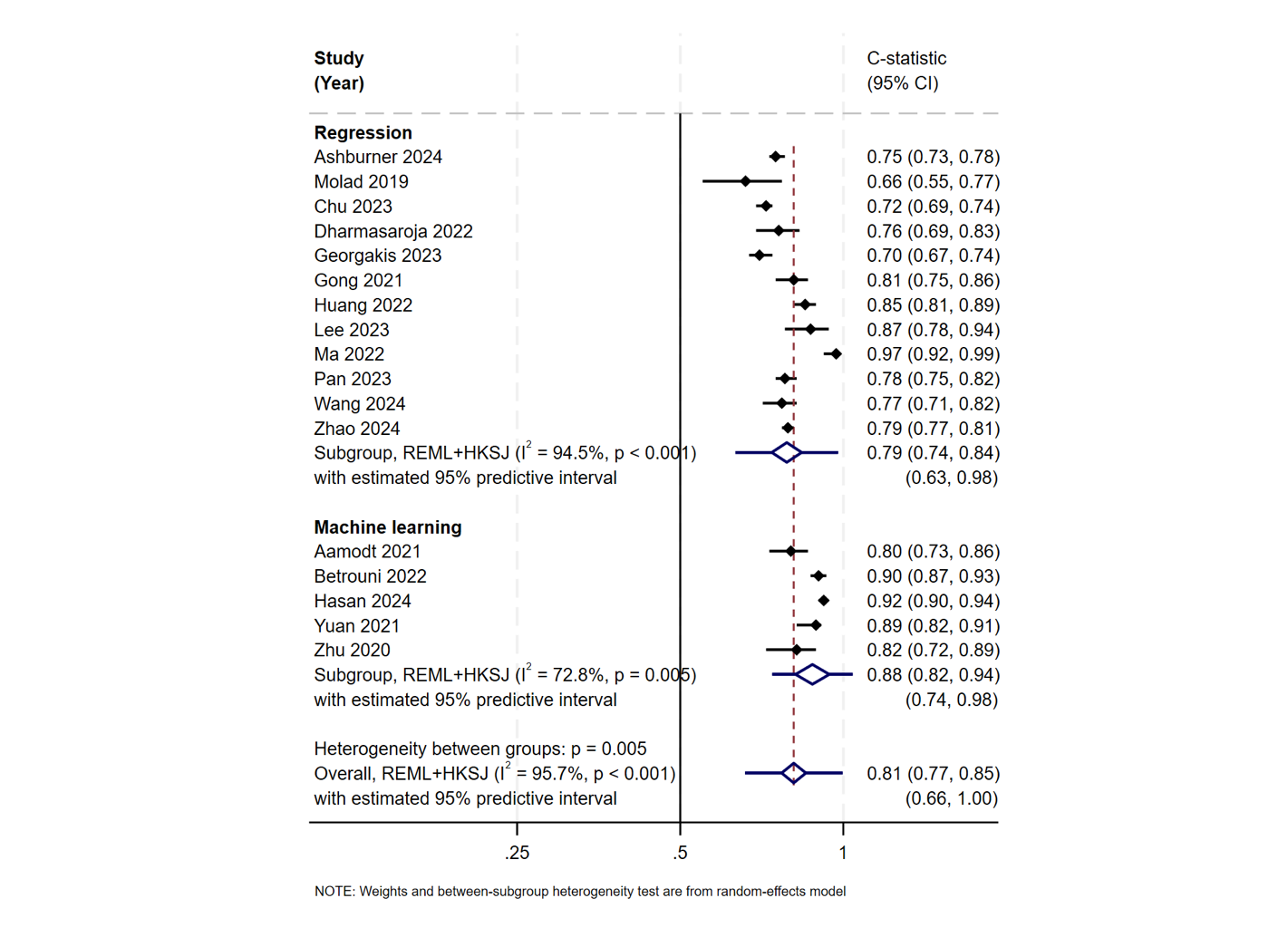
**

**Supplementary Figure 2: Publication Bias for Post-Stroke Cognitive Impairment**

**
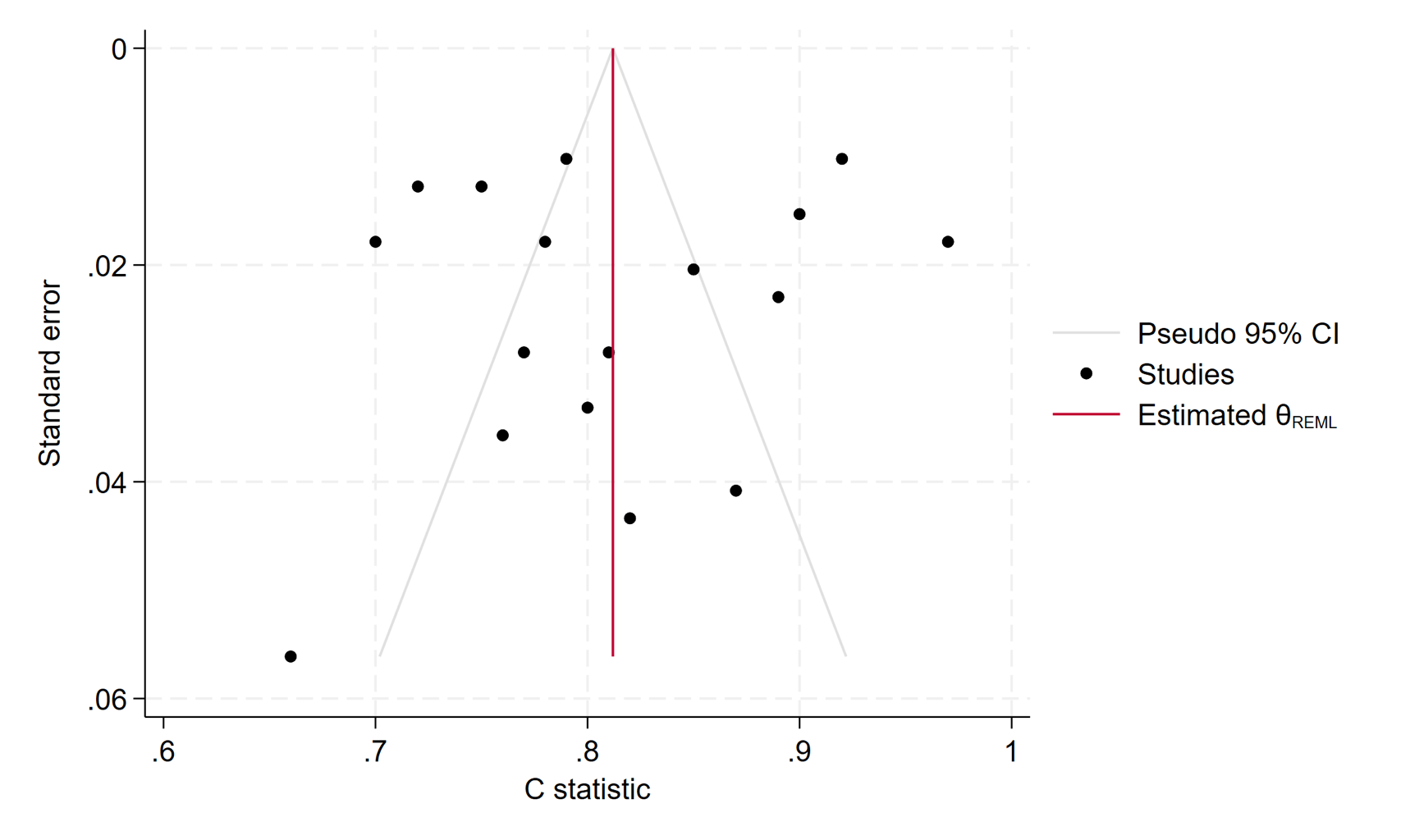
**

**Supplementary Table 3 Univariate meta-regression analysis for exploring potential source of heterogeneity in post-stroke cognitive impairment outcome**

| **Variable** | **Coefficient** | **Std. Err.** | **95% CI** | **P-value** |
| --- | --- | --- | --- | --- |
| **Age (years)** | **0.0031175** | **0.0057527** | **-0.0081576 to 0.0143925** | **0.588** |
| **Sample size** | **-0.000014** | **0.0000225** | **-0.000058 to 0.00003** | **0.532** |
| **Observed number of events** | **-0.0001162** | **0.000122** | **-0.0003553 to 0.0001228** | **0.341** |
| **Follow-up time (years)** | **0.0053045** | **0.0063198** | **-0.007082 to 0.0176911** | **0.401** |

**Supplementary Figure 3: Bubble plots for visual inspection of potential source of heterogeneity in post-stroke cognitive impairment outcome**

**Supplementary Table 4: GRADE Assessment of All Studies**

| **Outcomes** | **No. of:**   - **Studies** - **Prediction tools** - **Participants** - **Outcomes** | **Discrimination (range)**  **Calibration (range)** | **A** | **B** | **C** | **D** | **E** | **Certainty of the Evidence (GRADE)** |
| --- | --- | --- | --- | --- | --- | --- | --- | --- |
| Post Stroke Cognitive Impairment | 24  38  13871  1833 | 0.67-0.97  Not reported |  | x | x |  | x | Low |
| Delirium | 6  10  2793  663 | 0.73-0.90  Not reported |  | x | x |  | x | Low |

A=Risk of bias

B=Inconsistency Downgraded for both outcomes due to heterogeneity

C=Imprecision Downgraded for both outcomes due to range of values

D=Indirectness

E=Publication bias Downgraded for both outcomes due to lack of pre-registered protocols

**Supplementary Table 5: Post-Stroke Cognitive Impairment, Dementia Delirium Risk Models from Updated Search**

| **Study** | **Features (n)** | **Outcome** | **Ascertainment of Cognition** | **Timepoint of Outcome Assessment** | **Participants with Outcome, N (%)** | **Type of Model** | **Discrimination** | **Calibration** | **Validation** |
| --- | --- | --- | --- | --- | --- | --- | --- | --- | --- |
| ***Post-Stroke Cognitive Impairment*** | | | | | | | | | |
| *Logistic Regression* | | | | | | | | | |
| Ma 2025(1) | Age, education, global cortical atrophy, white matter hyperintensities, chronic cortical infarcts and intracranial stenosis | Post-stroke cognitive impairment | MoCA | 6 months | 321 (39.2%) | Logistic | AUC 0.87 (0.69-0.92) | Not reported | AUC=0.74 (0.71-0.80) |
| Wang 2025(2) | Age, education level, infarction in critical brain regions, LDL-C, white matter hyperintensities, brain atrophy | Post-stroke cognitive impairment | MoCA | 6 months | 143 (49.3%) | Logistic | Development AUC 0.89 | The accuracy of the nomogram prediction model was assessed using a calibration curve. The risk nomogram prediction model developed in this study demonstrates good predictive accuracy | Internal validation AUC=0.94 |
| Wei 2024a(3) | Smoking, alcohol consumption, female sex, educational attainment that is low, the NIHSS score upon admission, stroke progression, diabetes mellitus, a history of atrial fibrillation, stroke localization, homocysteine, lipoprotein-associated phospholipase | Post-stroke cognitive impairment | MoCA | 6 months | Not reported separately for development cohort. Overall for development/internal validation n=174 (45.1%) | Logistic | Development cohort AUC 0.90 (0.85-0.94) | Development: Hosmer-Lemeshow chi-squared statistic of 3.2885 and a *p*-value of 0.915  Internal validation: Hosmer-Lemeshow chi-squared value of 15.04 and a *p*-value of 0.06. | Internal validation cohort (n=193) AUC=0.85 (0.79-0.90)  External validation (n=187), AUC=0.85 (0.79-0.90)  Hosmer-Lemeshow chi-squared value of 11.6 and a corresponding *p*-value of 0.17. |
| Wei 2024b(4) | Sex, Age, history of stroke, NIHSS, education levels and fibrinogen | Cognitive impairment | MoCA | 7-14 days | 225 (56.5%) | Logistic | AUC: 0.76 (0.71-0.81) | The model’s predictions were closely aligned with the ideal model, indicating strong consistency | N/A |
| Yang 2025(5) | Smoking, alcohol consumption, female gender, low education level, NIHSS score at admission, stroke progression, high systolic blood pressure, diabetes, atrial fibrillation, coronary heart disease, low-density lipoprotein cholesterol, β2-microglobulin, and Lp-PLA2 | PSCI | MoCA | 6 months | 174 (45.1%) | Logistic Regression | AUC 0.86 (0.82-0.91) | Development: Hosmer-Lemeshow chi-squared value of 8.5443 and *P*=0.3822  Internal validation: Hosmer-Lemeshow chi-squared value in the validation set was 3.7208, with *P*=0.8814. | Internal validation AUC=0.81 (0.73-0.89) |
| Zhao 2025(6) | Model 1: Age, ferritin, previous stroke and MoCA  Model 2: Age, ferritin, previous stroke and education | Cognitive impairment | MoCA | Up to 24 months | 76 (29.8%) | Logistic regression | Model 1: AUC=0.97 (0.96-0.99)  Excluding patients with recurrent stroke, the AUC for model  1 0.97 (0.96–0.99)  Model 2: AUC=0.77 (0.71-0.83)  Excluding patients with recurrent stroke, the AUC for model  2 AUC= 0.78 (0.71–0.84) | The  calibration plots overlapped with the ideal line, indicating that the  predicted models sufficiently agreed with the actual observations | NA |
| *Machine Learning* | | | | | | | | | |
| Wei 2024c(7) | Stroke sites (brain stem, temporal lobe, subarachnoid space, outer capsule, and Island leaves), age, Lesion (right lesion), cerebral hemorrhage, high-sensitive CRP. | Post-stroke dementia | Not stated | Not stated | 345 (64.0%) | Logistic regression (LR); Extreme gradient boosting (XGB); Decision tree (DT); support vector machine (SVM); k-nearest neighbors (KNN); random forest (RF); multilayer perceptron (MLP); elastic net (EN) | XGB model had the highest AUC (0.7287), followed by RF (0.7285), KNN (0.7113), MLP (0.7082), EN (0.7033), LR (0.7022), DT (0.6502), and SVM (0.6098) | N/A | N/A |
| Zhang 2024(8) | Right hemisphere, urine glucose, urine protein, age, left hemisphere, infarct volume, blood glucose, free fatty acids, white blood cells | Post-stroke cognitive impairment | MMSE and the Chinese Dementia and Cognitive Impairment Diagnosis and Treatment Guideline | 3 days | 40 (41.7%) | SVM, Gaussian Naïve Bayes, LR | SVM AUC 0.82, Gaussian Naïve Bayes AUC =0.78 and LR AUC -0.86 | Not formally reported but on visual inspection of figure 3b, poor agreement between observed and predicted values. | NA |
| ***Delirium*** | | | | | | | | | |
| *Logistic Regression* | | | | | | | | | |
| Berger 2025(9) | Previous delirium, chronic alcohol consumption, infection, male sex, age (>70), vision/hearing impairment, non-lacunar stroke, NIHSS >7 on admission | Delirium | ICD-10 Diagnostic code | Not stated | 398 (6.5%) | Logistic regression | Derivation cohort: AUC=0.72 (0.69-0.75) | The calibration plot shows a good agreement  between the predicted and the observed delirium risk in the  validation cohort | Internal Validation cohort: AUC=0.72 (0.70-0.74) |
| Cai 2025(10) | Coronary heart disease, indwelling catheter, physical restraint, neutrophil-to-lymphocyte ratio | Delirium | Chinese version of 3 min Diagnostic Interview for  Confusion Assessment Method | Up to 3 days; end of observation was the occurrence of delirium, transfer, discharge or death | 105 (21.1%) in the whole cohort | Logistic regression | AUC (train group)=0.89 | The calibration curves of this  nomogram in the train group and the test  group were both close to the standard line. The result of Hosmer-Lemeshow goodness-of-fit test was χ2 = 3.903,  P = 0.917 in the train group, and χ2 = 7.165, P = 0.619 in the test group. These results indicate  that this nomogram had a good consistency between  the predictive and the actual incidence of PSD  occurrence. | AUC (test group)=0.87 |
| Cui 2025(11) | Age, visual impairment, post-stroke infection, NIHSS score and physical restraint | Post-stroke Delirium | Confusion assessment method for the intensive care unit | Till the end of hospital treatment | 141 (28.1%) | Logistic regression | AUC (modelling group)=0.92 | The Hosmer-Lemeshow goodness-of-fit test yielded a p . .646,  indicating good consistency between the nomogram model and  actual risk | AUC (internal validation cohort) = 0.90 |
| *Machine Learning* | | | | | | | | | |
| Kim 2025(12) | Age, sex (male), alcohol, NIHSS, HbA1c, prothrombin time, D-dimer, Hemoglobin, body temperature, heart rate, respiratory rate, oxygen saturation, systolic blood pressure, diastolic blood pressure | Delirium | Confusion assessment method for the intensive care unit or Intensive Care delirium Screening checklist | 16 hours | 84 (20.0%) | Logistic regression  Random Forest  lightGBM  SVM  XGBoost | Logistic regression AUC=0.80  Random Forest AUC=0.75  lightGBM AUC=0.75  SVM AUC=0.78  XGBoost AUC=0.76 | NA | Temporal validation: When assessing delirium occurrence against the matched non-delirium group, the AUROC was 0.78.  Evaluating over the entire duration of the ICU stay yielded an AUROC of 0.68 |

**References**

1. Ma N, Zhao Y, Meng X, Huang Y, Ma J, Liu X, et al. Development and Validation of a Clinical Model (SHACEA) for Post-stroke Cognitive Impairment Prognosis Occurred at Acute Phase and Last to 6 Months. Mol Neurobiol. 2025.

2. Wang M, Yang M, ZHan Z, Liang Y, Ju W. Establishment and evaluation of a risk predictive model for post-stroke cognitive impairment. Chinese Journal of Neurology. 2025;58(1):26-35.

3. Wei M, Zhu X, Yang X, Shang J, Tong Q, Han Q. Development and validation of a novel model to predict post-stroke cognitive impairment within 6 months after acute ischemic stroke. Frontiers in neurology. 2024;15:1451786.

4. Wei C, Zhai W, Zhao P, Sun L. Plasma fibrinogen as a potential biomarker of cognitive impairment after acute ischemic stroke. Scientific reports. 2024;14(1):32120.

5. Yang X, Wei M, Chen P, Shen J, Tong Q, Tian X, et al. Predicting poststroke cognitive impairment after acute ischemic stroke based on admission characteristics. Journal of stroke and cerebrovascular diseases : the official journal of National Stroke Association. 2024;33(12):108082.

6. Zhao P, Shi L, Zhang G, Wei C, Zhai W, Shen Y, et al. Development and internal validation of a nomogram for predicting cognitive impairment after mild ischemic stroke and transient ischemic attack based on cognitive trajectories: a prospective cohort study. Front Aging Neurosci. 2025;17:1427737.

7. Wei Z, Li M, Zhang C, Miao J, Wang W, Fan H. Machine learning-based predictive model for post-stroke dementia. BMC Med Inform Decis Mak. 2024;24(1):334.

8. Zhang J, Kong Z, Hong S, Zhang Z. Machine Learning-Based Model for Prediction of Post-Stroke Cognitive Impairment in Acute Ischemic Stroke: A Cross-Sectional Study. Neurol India. 2024;72(6):1193-8.

9. Berger N, Kramer D, Schrempf M, Hofer E, Pichler A, Fandler-Höfler S, et al. Predicting delirium in acute ischemic stroke: the PREDELIS score. J Neurol. 2025;272(6):391.

10. Cai X, Yu X, Qin J, Zhou K, Li Z, Zhang J, et al. Development and validation of a nomogram for delirium in the old ischaemic stroke patients. Psychogeriatrics. 2025;25(2):e13247.

11. Cui C, Han G, Wang Y, Zhao B, Li Q. Development and Validation of a Nomogram for Predicting Model for Delirium After Stroke. Asian Nurs Res (Korean Soc Nurs Sci). 2025;19(2):113-9.

12. Kim H, Kim M, Kim DY, Seo DG, Hong JM, Yoon D. Prediction of delirium occurrence using machine learning in acute stroke patients in intensive care unit. Front Neurosci. 2024;18:1425562.

**Supplementary Table 6 : Excluded Studies and Reason for Exclusion**

| **Title** | **Authors** | **Journal** | **Exclusion Reason** |
| --- | --- | --- | --- |
| Progression of cognitive decline before and after incident stroke | Zheng, Fanfan; Yan, Li; Zhong, Baoliang; Yang, Zhenchun; Xie, Wuxiang | Neurology | Wrong patient population |
| Relationships of Low Serum Levels of Interleukin-10 With Poststroke Anxiety and Cognitive Impairment in Patients With Clinical Acute Stroke | Z.-J., Ying; Y.-Y., Huang; M.-M., Shao; C.-H., Chi; M.-X., Jiang; Y.-H., Chen; Y., Chen; M.-X., Sun; Y.-Y., Zhu; X., Li | Journal of Clinical Neurology (Korea) | Single component |
| Plasma Neurofilament Light Chain as a Predictive Biomarker for Post-stroke Cognitive Impairment: A Prospective Cohort Study | Z., Wang; R., Wang; Y., Li; M., Li; Y., Zhang; L., Jiang; J., Fan; Q., Wang; D., Yang | Frontiers in Aging Neuroscience | Single component |
| Risk stratification and predictive modeling of postoperative delirium in chronic subdural hematoma | Yang, Xuan; Regmi, Moksada; Wang, Yingjie; Liu, Weihai; Dai, Yuwei; Liu, Shikun; Lin, Guozhong; Yang, Jun; Ye, Jingyi; Yang, Chenlong | Neurosurgical review | Wrong patient population |
| Association between serum cystatin C level and post-stroke cognitive impairment in patients with acute mild ischemic stroke | Yan, Xu; Chen, Huan; Shang, Xiu-Li | Brain and behavior | Wrong intervention |
| Development and validation of an interpretable machine learning model-Predicting mild cognitive impairment in a high-risk stroke population | Yan, Feng-Juan; Chen, Xie-Hui; Quan, Xiao-Qing; Wang, Li-Li; Wei, Xin-Yi; Zhu, Jia-Liang | Frontiers in Aging Neuroscience | Wrong patient population |
| Machine learning prediction for poststroke delirium using clinical and brain-regional characteristics of acute ischemic stroke patients | Y.K., Hyon; H.W., Yang; K.W., Jeon; M., Lee; T.J., Ha; J.W., Shin; J.L., Kim; J., Kim | European Stroke Journal | Abstract only |
| Multiplex array analysis of serum cytokines offers minimal predictive value for cognitive function in the subacute phase after stroke | Y., Zhang; H., Song; J., Wang; X., Xi; P., Cefalo; L.J., Wood; X., Luo; Q.M., Wang | Frontiers in Neurology | Wrong outcomes |
| USING MEMTRAX MEMORY TEST TO DETECT POST-STROKE COGNITIVE IMPAIRMENT AFTER ISCHEMIC STROKE | X., Liu; J., Zhang; M.F., Bergeron; J.W., Ashford; X., Zhou; L., Zhong; X., Zhao; X., Chen | European Stroke Journal | Abstract only |
| Comparative Study of Two Short-Form Versions of the Montreal Cognitive Assessment for Screening of Post-Stroke Cognitive Impairment in a Chinese Population | Wei, Jingjing; Jin, Xianglan; Chen, Baoxin; Liu, Xuemei; Zheng, Hong; Guo, Rongjuan; Liang, Xiao; Fu, Chen; Zhang, Yunling | Clinical interventions in aging | Wrong intervention |
| The montreal cognitive assessment and mini-mental state examination visuoexecutive subtests in acute ischemic stroke patients and their correlations with demographic and clinical factors | Wang, Wei; Dong, Fang-Ming; Shao, Kai; Guo, Shang-Zun; Zhao, Zhong-Min; Yang, Yi-Ming; Song, Ya-Xue; Wang, Jian-Hua | Acta neurologica Belgica | Wrong outcomes |
| Using Fractional Amplitude of Low-Frequency Fluctuations and Functional Connectivity in Patients With Post-stroke Cognitive Impairment for a Simulated Stimulation Program | Wang, Sirui; Rao, Bo; Chen, Linglong; Chen, Zhuo; Fang, Pinyan; Miao, Guofu; Xu, Haibo; Liao, Weijing | Frontiers in Aging Neuroscience | Wrong patient population |
| Serum Galectin-3 as a Potential Predictive Biomarker Is Associated with Poststroke Cognitive Impairment | Wang, Qian; Wang, Kai; Ma, Yihong; Li, Simin; Xu, Yuzhen | Oxidative medicine and cellular longevity | Wrong outcome |
| Stroke to Dementia Associated with Environmental Risks-A Semi-Markov Model | Wang, Kung-Jeng; Lee, Chia-Min; Hu, Gwo-Chi; Wang, Kung-Min | International journal of environmental research and public health | Wrong study design |
| Analysis of correlation between plasma low density lipoprotein cholesterol, homocysteine and cognitive function in patients with cerebral small vascular disease | W., Wang; Z., Shen; F., Li; Y., Sun; W., Sun | Chinese Journal of Cerebrovascular Diseases | Abstract only |
| Predictors of Cognitive Impairments in Patients with First-Ever and Recurrent Stroke | TOMBAK, Yasemin; G√úR√áAY, Eda; KARAAHMET, √ñzg√ºr Zeliha; UMAY, Ebru; TAMKAN, Arif; √áAKCI, Ayt√ºl | Journal of Physical Medicine & Rehabilitation Sciences | Wrong intervention |
| Cognitive predictors of a performance-based measure of instrumental activities of daily living following stroke | Tiznado, Denisse; Clark, Jillian M R; McDowd, Joan | Topics in stroke rehabilitation | Wrong study design |
| Neuroplasticity vs. cognitive prediction models in acute ischaemic stroke: limitations must be remembered | Tibocha-Gordon, Ingrid Xiomara; Morales-Ospina, Vanessa; Vergara-Jacome, Yudex Abraham; Lozada-Martinez, Ivan David | Neurologia i neurochirurgia polska | Editorial |
| Post-stroke cognitive complaints and normal moca scores: A possible role for machine learning-augmented cognitive screening | T.S., Field; M., Zhang; H., Jang; A.D., Rebchuk; G., Carenini | Stroke | Abstract only |
| Stroke prediction and the future of prognosis research | T.J., Quinn; B.A., Drozdowska | Nature Reviews Neurology | Not primary research |
| Early Cognitive Impairment after Minor Stroke: Associated Factors and Functional Outcome | Suda, Satoshi; Nishimura, Takuya; Ishiwata, Akiko; Muraga, Kanako; Aoki, Junya; Kanamaru, Takuya; Suzuki, Kentaro; Sakamoto, Yuki; Katano, Takehiro; Nishiyama, Yasuhiro; Mishina, Masahiro; Kimura, Kazumi | Journal of stroke and cerebrovascular diseases : the official journal of National Stroke Association | Wrong study design |
| The Impact of Age and Severity on Dementia After Traumatic Brain Injury: A Comparison Study | Stopa, Brittany M; Tahir, Zabreen; Mezzalira, Elisabetta; Boaro, Alessandro; Khawaja, Ayaz; Grashow, Rachel; Zafonte, Ross D; Smith, Timothy R; Gormley, William B; Izzy, Saef | Neurosurgery | Wrong patient population |
| Delirium in an Acute Stroke Setting, Occurrence, and Risk Factors | Shaw, Robert; Drozdowska, Bogna; Taylor-Rowan, Martin; Elliott, Emma; Cuthbertson, Gillian; Stott, David J; Quinn, Terence J | Stroke | Wrong study design |
| Prediction of post-stroke cognitive impairment by Montreal Cognitive Assessment (MoCA) performances in acute stroke: comparison of three normative datasets | Salvadori, Emilia; Cova, Ilaria; Mele, Francesco; Pomati, Simone; Pantoni, Leonardo | Aging clinical and experimental research | Wrong intervention |
| The Influence of Stroke Location on Cognitive and Mood Impairment. A Voxel-Based Lesion-Symptom Mapping Study | Sagnier, Sharmila; Munsch, Fanny; Bigourdan, Antoine; Debruxelles, Sabrina; Poli, Mathilde; Renou, Pauline; Olindo, Stephane; Rouanet, Francois; Dousset, Vincent; Tourdias, Thomas; Sibon, Igor | Journal of stroke and cerebrovascular diseases : the official journal of National Stroke Association | Wrong intervention |
| Validation of a New Cognitive Screening Method for Stroke Patients | Saar, Katri; Nyrkko, Hannu; Tolvanen, Asko; Kuikka, Pekka; Poutiainen, Erja; Aro, Tuija | Behavioural neurology | Wrong study design |
| Identifying Stroke Patients At Risk For Cognitive Impairment And Dementia Using Electronic Health Record Data And Machine Learning | S.M.S., Hasan; T., Su; J., Saurman; F.B., Nahab; X., Hu | Stroke | Abstract only |
| The severity of cognitive impairment in lacunar stroke, depending on the location of the lesion in right-handed patients | S., Shokhimardonov; S., Khudjanov; S., Kuzieva; F., Shermukhameedova; K., Daminova | International Journal of Stroke | Abstract only |
| Platelet procoagulant potential and sex are predictive of cognitive impairment at 12 months after lacunar stroke | S., Ramirez-Salazar; A.C., Kirkpatrick; A., Vicent; B., Ray; J., Scott; G., Dale; C., Prodan | Neurology | Abstract only |
| MRI Predictors of Cognitive Outcome After Small Vessel Disease Stroke | S., Hassani; D., Koltai; T., Amrhein; S., Unnithan; H., Al-Khalidi; C., Bushnell; L., Goldstein; N., El Husseini | Neurology | Abstract only |
| Clinical and demographic characteristics associated with cognitive recovery during stroke inpatient rehabilitation | S., Aggarwal; X., Zhang; D., Parker; S., Taleb; J., Wozny; N., Wewior; C., Silos; S., Paladugu; L., Ibrahim; M.B., Tariq; B., Hsieh; D., Norris; M., Verduzco-Gutierrez; S.I., Savitz | Stroke | Conference |
| Cognitive Impairment and Dementia After Stroke: Design and Rationale for the DISCOVERY Study | Rost, Natalia S; Meschia, James F; Gottesman, Rebecca; Wruck, Lisa; Helmer, Karl; Greenberg, Steven M | Stroke | Protocol |
| What neuropsychological functions best discriminate performance in adults post-stroke? | Rodrigues, Jaqueline de Carvalho; Machado, Wagner de Lara; da Fontoura, Denise Ren; Almeida, Andrea Garcia; Brondani, Rosane; Martins, Sheila Ouriques; Ruschel Bandeira, Denise; Salles, Jerusa Fumagalli de | Applied neuropsychology. Adult | Wrong study design |
| Predicting cognitive impairment in cerebrovascular disease using spoken discourse production | Roberts, Angela; Aveni, Katharine; Basque, Shalane; Orange, Joseph B; McLaughlin, Paula; Ramirez, Joel; Troyer, Angela K; Gutierrez, Stephanie; Chen, Angie; Bartha, Robert; Binns, Malcolm A; Black, Sandra E; Casaubon, Leanne K; Dowlatshahi, Dar; Hassan, Ayman; Kwan, Donna; Levine, Brian; Mandzia, Jennifer; Sahlas, Demetrios J; Scott, Christopher J. M; Strother, Stephen; Sunderland, Kelly M; Symons, Sean; Swartz, Richard | Topics in Language Disorders | Wrong study design |
| Screening for cognitive impairment with the Montreal Cognitive Assessment in Spanish patients with minor stroke or transient ischaemic attack | Ramirez-Moreno, J M; Bartolome Alberca, S; Munoz Vega, P; Guerrero Barona, E J | Neurologia | Wrong study design |
| Dementia risk following ischemic stroke: A systematic review and meta-analysis of factors collected at time of stroke diagnosis | R., Waziry; J., Claus; A., Hofman | Stroke | Wrong study design |
| Depressive-, cognitive-or stroke-related risk factors of post-stroke depression: Which one could better help clinicians and patients? | R., Perrain; D., Calvet; V., Guiraud; L., Mekaoui; J.-L., Mas; P., Gorwood | Neuropsychiatric Disease and Treatment | Wrong outcome |
| Duration and Efficiency of Combined versus Isolated Aerobic Training Interventions in Post-Stroke Cognition: A Systematic Review | R., Maeneja; I.S., Ferreira; A.M., Abreu | Portuguese Journal of Public Health | Not primary research |
| Unsupervised machine learning model to predict cognitive impairment in subcortical ischemic vascular disease | Qin, Qi; Qu, Junda; Yin, Yunsi; Liang, Ying; Wang, Yan; Xie, Bingxin; Liu, Qingqing; Wang, Xuan; Xia, Xinyi; Wang, Meng; Zhang, Xu; Jia, Jianping; Xing, Yi; Li, Chunlin; Tang, Yi | Alzheimer's & dementia : the journal of the Alzheimer's Association | Wrong patient population |
| Plasma exosome proteomics reveals the pathogenesis mechanism of post-stroke cognitive impairment | Qi, Baoyun; Kong, Lingbo; Lai, Xinxing; Wang, Linshuang; Liu, Fei; Ji, Weiwei; Wei, Dongfeng | Aging | Wrong study design |
| The validity of the Montreal cognitive assessment (MoCA) for the screening of vascular cognitive impairment after ischemic stroke | Potocnik, Jure; Ovcar Stante, Klavdija; Rakusa, Martin | Acta neurologica Belgica | Wrong outcome |
| DELIRIUM IN ACUTE STROKE PATIENTS: DEVELOPMENT OF A NEW PREDICTION SCORE | P., Kremer; J., Wischmann; A., Becker-Pennrich; L., Hinske; L., Kellert | European Stroke Journal | Abstract only |
| Cognitive decline before and after a first-ever stroke in Africans | Ojagbemi, Akin; Bello, Toyin; Owolabi, Mayowa; Baiyewu, Olusegun | Acta neurologica Scandinavica | Not stated PSCI as an exclusion |
| Predicting dementia in cerebral small vessel disease using an automatic diffusion tensor image segmentation technique | O.A., Williams; E.A., Zeestraten; P., Benjamin; C., Lambert; A.J., Lawrence; A.D., Mackinnon; R.G., Morris; H.S., Markus; T.R., Barrick; R.A., Charlton | Stroke | Single component |
| VasCog Screen test: Sensitive in detecting cognitive impairment in patients who had a stroke or with heart failure | N.Y.C., Chen; M.Y.L., Tan; J., Xu; L., Zuo; Y., Dong | Stroke and Vascular Neurology | Wrong study design |
| Strategic infarct locations for post-stroke cognitive impairment: A large-scale multicenter lesion-symptom mapping study | N.A., Weaver | International Journal of Stroke | Abstract only |
| Test Accuracy of the Montreal Cognitive Assessment in Screening for Early Poststroke Neurocognitive Disorder: The Nor-COAST Study | Munthe-Kaas, Ragnhild; Aam, Stina; Saltvedt, Ingvild; Wyller, Torgeir Bruun; Pendlebury, Sarah T; Lydersen, Stian; Ihle-Hansen, Hege | Stroke | Wrong study design |
| Examining the Associations between Post-Stroke Cognitive Function and Common Comorbid Conditions among Stroke Survivors | Morrison, Helena W; White, Melissa M; Rothers, Janet L; Taylor-Piliae, Ruth E | International journal of environmental research and public health | Wrong study design |
| Factors associated with cognitive improvement in subacute stroke survivors | Mori, Naoki; Otaka, Yohei; Honaga, Kaoru; Matsuura, Daisuke; Kondo, Kunitsugu; Liu, Meigen; Tsuji, Tetsuya | Journal of rehabilitation medicine | Wrong patient population |
| Post-Stroke Cognitive Impairment is Frequent After Infra-Tentorial Infarct | Moliis, Henrik; Jokinen, Hanna; Parkkonen, Eeva; Kaste, Markku; Erkinjuntti, Timo; Melkas, Susanna | Journal of stroke and cerebrovascular diseases : the official journal of National Stroke Association | Wrong study design |
| Domain-specific cognitive impairment 6 months after stroke: The value of early cognitive screening | Milosevich, Elise T; Moore, Margaret J; Pendlebury, Sarah T; Demeyere, Nele | International journal of stroke : official journal of the International Stroke Society | Wrong intervention |
| Examining the associations between post-stroke cognitive function and recurrent stroke risk factors that include co-morbid conditions | McElroy, Melissa Michaels | Dissertation Abstracts International: Section B: The Sciences and Engineering | Not primary research |
| Risk of recurrent stroke and dementia following acute stroke by changes in kidney function: results from the Perindopril Protection Against Recurrent Stroke Study | Maeda, Toshiki; Woodward, Mark; Jun, Min; Sakamoto, Yuki; Chen, Xiaoying; Matsushita, Kunihiro; Mancia, Giuseppe; Arima, Hisatomi; Anderson, Craig S; Chalmers, John; Harris, Katie | Journal of hypertension | Wrong outcome |
| Association Between Education Level and PostStroke Cognitive Decline - A Pooled Cohort Analysis of Four Cohorts | M.V., Springer; R.T., Whitney; W., Ye; E.M., Briceno; A.L., Gross; H.J., Aparicio; A.S., Beiser; J.F., Burke; M.S., Elkind; R.A., Ferber; B.J., Giordani; R.F., Gottesman; R.A., Hayward; V.J., Howard; A.S., Kollipara; S., Koton; R.M., Lazar; W.T., Longstreth; S.T., Pendlebury; J.B., Sussman; E.L., Thacker; D.A., Levine | Stroke | Abstract only |
| Cognitive dysfunctions long-term after stroke occurrence: A study in patients from register of Ignace Deen teaching hospital Conakry-Guinea | M.C., Diallo; P.K., N'Go; A.O.T., Ahami; F.A., Cisse; V.O., Obayagbona | Activitas Nervosa Superior Rediviva | Wrong study design |
| The association between decreased cerebral blood flow in transient ischemic attack patients and cognition | M., Reid; C., McDougall; N., Forkert; R., Frayne; S., Coutts; R.G., Sah; C.D., D'Esterre; P., Barber | Stroke | Abstract only |
| Prediction of cognitive decline after intracerebral haemorrhage using total small vessel disease score | M., Pasi; L., Sugita; L., Xiong; A., Charidimou; G., Boulouis; T., Pongpitakmetha; S.D., Singh; C., Kourkoulis; K., Schwab; S.M., Greenberg; C.D., Anderson; M.E., Gurol; A., Viswanathan; A., Biffi | European Stroke Journal | Abstract only |
| CEREBRAL SMALL VESSEL DISEASE BURDEN AND COGNITIVE AND FUNCTIONAL OUTCOMES AFTER STROKE: A MULTICENTER PROSPECTIVE COHORT STUDY | M., Georgakis; R., Fang; M., During; F., Wollenweber; A., Dewenter; F., Bode; S., Stosser; C., Kindlein; P., Hermann; T., Liman; C., Nolte; L., Kerti; B., Ikenberg; K., Bernkopf; H., Poppert; W., Glanz; D., Janowitz; M., Wagner; K., Neumann; O., Speck; L., Dobisch; E., Duezel; B., Gesierich; K., Waegemann; M., Gortler; S., Wunderlich; M., Endres; I., Zerr; G., Petzold; M., Dichgans | European Stroke Journal | Wrong intervention |
| S100B is associated with the development of post stroke delirium in patients with acute ischemic stroke | M., Arnold; T., Honegger; J., Schweizer; V., Schutz; C., Inauen; T., Pokorny; L., Westphal; A., Bivic; A., Luft; K., Spanaus; A., Von Eckardstein; M., Katan | European Stroke Journal | Abstract only |
| Short-term Trajectories of Poststroke Cognitive Function: A STROKOG Collaboration Study | Lo, Jessica W; Crawford, John D; Desmond, David W; Bae, Hee-Joon; Lim, Jae-Sung; Godefroy, Olivier; Roussel, Martine; Kohler, Sebastian; Staals, Julie; Verhey, Frans; Chen, Christopher; Xu, Xin; Chong, Eddie J; Kandiah, Nagaendran; Bordet, Regis; Dondaine, Thibaut; Mendyk, Anne-Marie; Brodaty, Henry; Traykov, Latchezar; Mehrabian, Shima; Petrova, Neli; Lipnicki, Darren M; Lam, Ben Chun Pan; Sachdev, Perminder S | Neurology | Not primary research |
| Structural Change of Gut Microbiota in Patients with Post-Stroke Comorbid Cognitive Impairment and Depression and Its Correlation with Clinical Features | Ling, Yi; Gu, Qilu; Zhang, Junmei; Gong, Tianyu; Weng, Xiongpeng; Liu, Jiaming; Sun, Jing | Journal of Alzheimer's disease : JAD | Wrong study design |
| Cerebral Small Vessel Disease Burden Is Associated With Accelerated Poststroke Cognitive Decline: A 1-Year Follow-Up Study | Liang, Yan; Chen, Yang-Kun; Liu, Yong-Lin; Mok, Vincent C. T.; Ungvari, Gabor S.; Chu, Winnie C. W.; Seo, Sang Won; Tang, Wai-Kwong; Xiang, Yu-Tao | Journal of Geriatric Psychiatry & Neurology | Wrong outcome |
| Association of the sarcopenia index with cognitive impairment in a middle-aged to older patients with acute ischemic stroke or transient ischemic attack: A multicenter cohort study | Li, Siqi; Yan, Hongyi; Pan, Yuesong; Zhang, Yumei | The journal of nutrition, health & aging | Wrong intervention |
| Prediction of post-stroke cognitive impairment using brain FDG PET: deep learning-based approach | Lee, Reeree; Choi, Hongyoon; Park, Kwang-Yeol; Kim, Jeong-Min; Seok, Ju Won | European journal of nuclear medicine and molecular imaging | Not stated PSCI as an exclusion |
| Association between Geriatric Nutritional Risk Index and Post-Stroke Cognitive Outcomes | Lee, Minwoo; Lim, Jae-Sung; Kim, Yerim; Lee, Ju Hun; Kim, Chul-Ho; Lee, Sang-Hwa; Jang, Min Uk; Oh, Mi Sun; Lee, Byung-Chul; Yu, Kyung-Ho | Nutrients | Wrong intervention |
| Analysis of risk factors for the development of cognitive dysfunction in patients with cerebral small vessel disease and the construction of a predictive model | L., Zhang; F., Gao; P., Hu; Y., Yao; Q., Zhang; Y., He; Q., Shang; Y., Zhang | Frontiers in Neurology | Wrong patient population |
| [The relationship between the development of post-stroke cognitive impairment and changes in the coagulation component of hemostasis] | Koltsov, I A; Shchukin, I A; Kovalenko, E A; Karpova, N S; Shilov, Y E; Brusov, O S | Zhurnal nevrologii i psikhiatrii imeni S.S. Korsakova | Unable to access |
| Latent profile analysis of cognitive decline and depressive symptoms after intracerebral hemorrhage | Keins, Sophia; Abramson, Jessica R; Castello, Juan Pablo; Pasi, Marco; Charidimou, Andreas; Kourkoulis, Christina; DiPucchio, Zora; Schwab, Kristin; Anderson, Christopher D; Gurol, M Edip; Greenberg, Steven M; Rosand, Jonathan; Viswanathan, Anand; Biffi, Alessandro | BMC neurology | Wrong patient population |
| Avoiding "toxic knowledge": The importance of framing personalized risk information in clinical decision-making | K.M., Kostick; J.S., Blumenthal-Barby | Personalized Medicine | Editorial |
| Predictability of cognitive functions on discharge destination in acute stroke patients | K., Stibrant Sunnerhagen; A., Ferrario; M., Reinholdsson; T., Abzhandadze | Neurorehabilitation and Neural Repair | Unable to access |
| Stroke Prognostic Scores and Data-Driven Prediction of Clinical Outcomes After Acute Ischemic Stroke | K., Matsumoto; Y., Nohara; H., Soejima; T., Yonehara; N., Nakashima; M., Kamouchi | Stroke | Wrong outcome |
| THE APPLICATION VALUE OF SERUM 3-NT AND NLRP3 IN THE DIAGNOSIS OF COGNITIVE IMPAIRMENT IN PATIENTS WITH ISCHEMIC WHITE MATTER LESIONS | J., Wei; X., Jiao; X., Gao; W., Qian | Acta Medica Mediterranea | Abstract only |
| Individual prediction and classification of cognitive impairment in patients with white matter lesions based on gray matter volume | J., Wang; C., Zhao; J., Wei; C., Li; X., Zhang; Y., Liang; Y., Zhang | Annals of Translational Medicine | Wrong study design |
| Uncovering Post-stroke Cognitive Impairment: A Retrospective Analysis at the University of Oklahoma Vascular Neurology Service | J., Swinton; C.B., Pinto; A.L.O., Santos; C., Owens; C., Gutierrez; B.A., Hill; K., Singleton; Z., Stuart; A., Yabluchanskiy; F.S., Velez | Neurology | Abstract only |
| INCIDENCE AND PROGNOSTIC INDICATORS OF DEMENTIA IN PATIENTS WITH TIA AND STROKE: THE POPULATION-BASED ROTTERDAM STUDY | J., Claus; B., Camiel; V., Elizabeth; M., Rosbergen; M., Vernooij; M.K., Ikram; A., Ikram; F., Wolters | European Stroke Journal | Abstract only |
| The role of sleep-disordered breathing on cognitive function after stroke | I., Filchenko; S., Duss; C., Bernasconi; M.H., Schmidt; C.L.A., Bassetti | Sleep Medicine | Abstract only |
| Nonlinguistic Cognition Functions of Mandarin Speakers With Poststroke Aphasia | Huang, Tzu-Jung; Chang, Ping-Hsin; Chiou, Hsinhuei Sheen; Hsu, Hsin-jen | American Journal of Speech-Language Pathology | Wrong outcome |
| The Assessment and Prediction of Prospective Memory after Stroke | Hogan, Christy; Cornwell, Petrea; Fleming, Jennifer; Shum, David H K | Journal of the International Neuropsychological Society : JINS | Wrong intervention |
| Risk Prediction of Cognitive Decline after Stroke | Hbid, Youssef; Fahey, Marion; Wolfe, Charles D A; Obaid, Majed; Douiri, Abdel | Journal of stroke and cerebrovascular diseases : the official journal of National Stroke Association | Wrong patient population |
| Predictors for Favorable Cognitive Outcome Post-Stroke: A-Seven-Year Follow-Up Study | Hagberg, Guri; Fure, Brynjar; Thommessen, Bente; Ihle-Hansen, Hakon; Oksengard, Anne-Rita; Nygard, Stale; Pendlebury, Sarah T; Beyer, Mona K; Wyller, Torgeir Bruun; Ihle-Hansen, Hege | Dementia and geriatric cognitive disorders | Wrong patient population |
| Prediction enhancement of the post-stroke delirium by adding of the brain lesional characteristics to the clinical features in acute ischemic stroke patients | H.S., Jeong; H.W., Yang; S.H., Lee; Y.K., Hyon; K., Jeon; M., Lee; T., Ha; J.L., Kim; S., Lee; J., Kim | Stroke | Abstract only |
| [Post-stroke cognitive deficits and dementia] | Gallucci, Laura; Umarova, Roza M | Therapeutische Umschau. Revue therapeutique | Unable to access |
| Improving diagnostic accuracy of the Montreal Cognitive Assessment to identify post-stroke cognitive impairment | Gallucci, Laura; Sperber, Christoph; Monsch, Andreas U; Kloppel, Stefan; Arnold, Marcel; Umarova, Roza M | Scientific reports | Wrong study design |
| Predicting dementia after stroke | G.J., Biessels | International Journal of Stroke | Conference |
| Predicting stroke outcome: Role of a biomarker panel | G.C., Jickling; T.L., Russo | Neurology | Editorial |
| Establishment and evaluation of a clinical prediction model for cognitive impairment in patients with cerebral small vessel disease | F., Zhu; J., Yao; M., Feng; Z., Sun | BMC Neuroscience | Wrong patient population |
| Traumatic Intracerebral Hemorrhage Confers a Higher Risk for Dementia than Spontaneous Intracerebral Hemorrhage | F., Radmanesh; Z., Tahir; T., Yahya; W., Li; H.A.E., Hassan; S., Snider; S., Izzy | Neurology | Abstract only |
| The influence of sleep apnoea syndrome on cognitive functions in patients with cerebral infarction | E.V., Serebrova | Siberian Medical Review | Wrong study design |
| Prediction of early post-stroke major neurocognitive disorder using support vector machines | E.B., Aamodt; T., Schellhorn; L., Apostolova; D.O., Svaldi; E., Stage; P.E., Logan; A.B., Sanjay; I., Saltvedt; M.K., Beyer | Stroke | Abstract only |
| Post-stroke reorganization of transient brain activity characterizes deficits and recovery of cognitive functions | E., Pirondini; N., Kinany; C.L., Sueur; J.C., Griffis; G.L., Shulman; M., Corbetta; D.V.D., Ville | NeuroImage | Wrong outcome |
| Prevalence and predictors of domain-specific cognitive impairment 6 months after stroke: the value of early cognitive screening | E., Milosevich; M., Moore; S., Pendlebury; N., Demeyere | International Journal of Stroke | Abstract only |
| Domain-specific cognitive impairments, mood and quality of life 6 months after stroke | E., Milosevich; A., Kusec; S.T., Pendlebury; N., Demeyere | Disability and rehabilitation | Wrong outcome |
| Cognitive predictors of memory impairment after stroke | E., Chan; S., Anderson; G., Banerjee; D., Turner; R., Simister; D., Werring; L., Cipolotti | European Stroke Journal | Conference |
| Neuroimaging improves the prediction of post-stroke major neurocognitive disorder | E., Aamodt; T., Schellhorn; E., Stage; A., Sanjay; P., Logan; D., Svaldi; L., Apostolova; I., Saltvedt; M., Beyer | Journal of the Neurological Sciences | Abstract only |
| Structural brain network measures are superior to vascular burden scores in predicting early cognitive impairment in post stroke patients with small vessel disease | Du, Jing; Wang, Yao; Zhi, Nan; Geng, Jieli; Cao, Wenwei; Yu, Ling; Mi, Jianhua; Zhou, Yan; Xu, Qun; Wen, Wei; Sachdev, Perminder | NeuroImage. Clinical | Wrong study design |
| Cardiovascular risk factors indirectly affect acute post-stroke cognition through stroke severity and prior cognitive impairment: a moderated mediation analysis | Drozdowska, Bogna A; Elliott, Emma; Taylor-Rowan, Martin; Shaw, Robert C; Cuthbertson, Gillian; Langhorne, Peter; Quinn, Terence J | Alzheimer's research & therapy | Not stated PSCI as an exclusion |
| Everyday Abilities Scale for India in Screening for Poststroke Dementia Among Young Stroke Survivors | Done, Indira P; Aghoram, Rajeswari; Narayan, Sunil K | Alzheimer disease and associated disorders | Wrong intervention |
| Dementia risk after transient ischaemic attack and stroke | Dichgans, Martin | The Lancet Neurology | Not primary research |
| Neighborhood Resources and Health Outcomes Among Stroke Survivors in a Population-Based Cohort | Delhey, Leanna M; Shi, Xu; Morgenstern, Lewis B; Brown, Devin L; Smith, Melinda A; Case, Erin C; Springer, Mellanie V; Lisabeth, Lynda D | Journal of the American Heart Association | Wrong study design |
| Validity of an enhanced EQ-5D-5L measure with an added cognitive dimension in patients with stroke | de Graaf, J A; Kuijpers, Mmt; Visser-Meily, Jma; Kappelle, L J; Post, Mwm | Clinical rehabilitation | Wrong outcome |
| Prestroke Physical Activity and Poststroke Cognitive Performance | Damsbo, Andreas Gammelgaard; Mortensen, Janne Kaergaard; Kraglund, Kristian Lundsgaard; Johnsen, Soren Paaske; Andersen, Grethe; Blauenfeldt, Rolf Ankerlund | Cerebrovascular diseases (Basel, Switzerland) | Single component |
| Dementia after Ischemic Stroke, from Molecular Biomarkers to Therapeutic Options | Dammavalam, Vikalpa; Rupert, Deborah; Lanio, Marcos; Jin, Zhaosheng; Nadkarni, Neil; Tsirka, Stella E; Bergese, Sergio D | International journal of molecular sciences | Not primary research |
| Cognitive Impairment in Patients with Stroke | D'Souza, Caitlin E; Greenway, Melanie R F; Graff-Radford, Jonathan; Meschia, James F | Seminars in neurology | Abstract only |
| Neuropsychological screening in the acute phase of cerebrovascular diseases | Cova, Ilaria; Mele, Francesco; Zerini, Federica; Maggiore, Laura; Cucumo, Valentina; Brambilla, Michela; Rosa, Silvia; Bertora, Pierluigi; Salvadori, Emilia; Pomati, Simone; Pantoni, Leonardo | Acta neurologica Scandinavica | Wrong outcome |
| Strategic white matter hyperintensity locations associated with post-stroke cognitive impairment: A multicenter study in 1568 stroke patients | Coenen, Mirthe; de Kort, Floor As; Weaver, Nick A; Kuijf, Hugo J; Aben, Hugo P; Bae, Hee-Joon; Bordet, Regis; Chen, Christopher Plh; Dewenter, Anna; Doeven, Thomas; Dondaine, Thibaut; Duering, Marco; Fang, Rong; van der Giessen, Ruben S; Kim, Jonguk; Kim, Beom Joon; de Kort, Paul Lm; Koudstaal, Peter J; Lee, Minwoo; Lim, Jae-Sung; Lopes, Renaud; van Oostenbrugge, Robert J; Staals, Julie; Yu, Kyung-Ho; Biessels, Geert Jan; Biesbroek, J Matthijs | International journal of stroke : official journal of the International Stroke Society | Wrong outcome |
| Serum TG/HDL-C level at the acute phase of ischemic stroke is associated with post-stroke cognitive impairment | Cheng, Yongqing; Zhu, Honghong; Chen, Jin; Li, Lei; Liu, Changxia; Gao, Yang; Sun, Dingming | Neurological sciences : official journal of the Italian Neurological Society and of the Italian Society of Clinical Neurophysiology | Single component |
| Improved Dementia Prediction in Cerebral Small Vessel Disease Using Deep Learning-Derived Diffusion Scalar Maps From T1 | Chen, Yutong; Tozer, Daniel; Li, Rui; Li, Hao; Tuladhar, Anil; De Leeuw, Frank Erik; Markus, Hugh S | Stroke | Wrong intervention |
| Sleep Parameters and Plasma Biomarkers for Cognitive Impairment Evaluation in Patients With Cerebral Small Vessel Disease | Chen, Xiaohan; Fang, Zhuo; Zhao, Yike; Cheng, Wenbin; Chen, Honglin; Li, Genru; Xu, Jin; Deng, Jiale; Cai, Xiao; Zhuang, Jianhua; Yin, You | The journals of gerontology. Series B, Psychological sciences and social sciences | Wrong patient population |
| Prevalence of post-stroke cognitive impairment and associated risk factors in Chinese stroke survivors | Chau, Janita Pak Chun; Lo, Suzanne Hoi Shan; Zhao, Jie; Choi, Kai Chow; Butt, Laveeza; Lau, Alexander Yuk Lun; Mok, Vincent Chung Tong; Kwok, Zoe Ching Man; Thompson, David R | Journal of the neurological sciences | Wrong outcome |
| PSICOICTUS: EVALUATION AND PROGNOSIS OF AFFECTIVE AND COGNITIVE DISORDERS AFTER MINOR STROKE | C.P., Priego; R., Mitjana; A., Regue; D., Vazquez; G., Mauri; G., Arque; F.P., Garcia | European Stroke Journal | Conference |
| History of stroke doubles the risk of dementia: The brain attack surveillance in Corpus Christi (BASIC)-cognitive project | C.J., Becker; W., Chang; S.G., Heeringa; E.M., Briceno; L., Morgenstern | Stroke | Abstract only |
| The role of microembolic signals and thrombophilia in predicting cognitive outcome in young ischemic stroke | C., Kulyk; A., Fattorello Salimbeni; A., Pieroni; P., Simioni; M., Vosko; C., Baracchini | International Journal of Stroke | Abstract only |
| Circulating circular RNAs as novel biomarkers and functional prediction for the early diagnosis in post-stroke cognitive impairment: A single-center prospective study in China | C., Chen; Q., Liu; J., Wang; X., Shen; Z., Cao; X., Zhang; Q., Chen; L., Yu; Z., Chu; Q., Fang | Journal of Stroke and Cerebrovascular Diseases | Wrong patient population |
| Structural connectivity-based predictors of cognitive impairment in stroke patients attributable to aging | Buckova, Barbora Rehak; Kala, David; Korenek, Jakub; Matuskova, Veronika; Kumpost, Vojtech; Svobodova, Lenka; Otahal, Jakub; Skoch, Antonin; Sulc, Vlastimil; Olserova, Anna; Vyhnalek, Martin; Jansky, Petr; Tomek, Ales; Marusic, Petr; Jiruska, Premysl; Hlinka, Jaroslav | PloS one | Wrong outcome |
| Post-stroke outcomes predicted from multivariate lesion-behaviour and lesion network mapping | Bowren, Mark; Bruss, Joel; Manzel, Kenneth; Edwards, Dylan; Liu, Charles; Corbetta, Maurizio; Tranel, Daniel; Boes, Aaron D | Brain : a journal of neurology | Wrong intervention |
| Texture Features of Magnetic Resonance Images: an Early Marker of Post-stroke Cognitive Impairment | Betrouni, Nacim; Yasmina, Moussaoui; Bombois, Stephanie; Petrault, Maud; Dondaine, Thibaut; Lachaud, Cedrick; Laloux, Charlotte; Mendyk, Anne-Marie; Henon, Hilde; Bordet, Regis | Translational stroke research | Wrong intervention |
| Differential Impact of Stroke on Cognitive Impairment in Mexican Americans and Non-Hispanic White Americans | Becker, Christopher J; Heeringa, Steven G; Chang, Wen; Briceno, Emily M; Mehdipanah, Roshanak; Levine, Deborah A; Langa, Kenneth M; Gonzales, Xavier F; Garcia, Nelda; Longoria, Ruth; Springer, Mellanie V; Zahuranec, Darin B; Morgenstern, Lewis B | Stroke | Wrong outcome |
| Predictors of post-stroke cognitive impairment at three-month following first episode of stroke among patients attended at tertiary hospitals in Dodoma, central Tanzania: A protocol of a prospective longitudinal observational study metadata | Baraka, Alphonce; Meda, John; Nyundo, Azan | PloS one | Protocol |
| Traumatic brain injury is associated with higher rates of dementia: A two-institution experience | B., Stopa; E., Mezzalira; A., Boaro; A., Khawaja; S., Izzy; W., Gormley | Journal of Neurosurgery | Wrong patient population |
| Association between plasma L-carnitine and the risk of cognitive impairment in patients with acute ischemic stroke | B., Che; Y., Zhang; C., Zhong | Atherosclerosis | Conference |
| Prediction of Cognitive Recovery After Stroke: The Value of Diffusion-Weighted Imaging-Based Measures of Brain Connectivity | Aben, Hugo P; De Munter, Leonie; Reijmer, Yael D; Spikman, Jacoba M; Visser-Meily, Johanna M A; Biessels, Geert Jan; De Kort, Paul L M | Stroke | Wrong outcome |
| Extent to Which Network Hubs Are Affected by Ischemic Stroke Predicts Cognitive Recovery | Aben, Hugo P; Biessels, Geert Jan; Weaver, Nick A; Spikman, Jacoba M; Visser-Meily, Johanna M A; de Kort, Paul L M; Reijmer, Yael D | Stroke | Wrong outcome |
| Informant questionnaire on cognitive decline in the elderly (IQCODE) for post-event dementia in tia and stroke | A., Van Nieuwkerk; S., Pendlebury; A., Koteci; P., Rothwell | International Journal of Stroke | Conference |
| Plasma parameters and risk factors of patients with post-stroke cognitive impairment | Wu, Ji-Xia; Xue, Jian; Zhuang, Lei; Liu, Chun-Feng | Annals of palliative medicine | Not stated PSCI as an exclusion |
| Predictors of Cognitive Functions After Stroke Assessed Using the Wechsler Adult Intelligence Scale: A Retrospective Study | Su, Wenlong; Li, Hui; Dang, Hui; Han, Kaiyue; Liu, Jiajie; Liu, Tianhao; Liu, Ying; Tang, Zhiqing; Lu, Haitao; Zhang, Hao | Journal of Alzheimer's disease : JAD | Not stated PSCI as an exclusion |
| Multiple biomarkers covering several pathways improve predictive ability for cognitive impairment among ischemic stroke patients with elevated blood pressure | Zhu, Zhengbao; Zhong, Chongke; Guo, Daoxia; Bu, Xiaoqing; Xu, Tan; Guo, Libing; Liu, Jiale; Zhang, Jintao; Li, Dong; Zhang, Jianhui; Ju, Zhong; Chen, Chung-Shiuan; Chen, Jing; He, Jiang; Zhang, Yonghong | Atherosclerosis | Not stated PSCI as an exclusion |
| Serum Rheumatoid Factor Levels at Acute Phase of Ischemic Stroke are Associated with Poststroke Cognitive Impairment | Zhu, Zhengbao; Chen, Lihua; Guo, Daoxia; Zhong, Chongke; Wang, Aili; Bu, Xiaoqing; Xu, Tan; Zhang, Jianhui; Ju, Zhong; Guo, Libing; Zhang, Jintao; Li, Dong; Chen, Chung-Shiuan; Chen, Jing; Zhang, Yonghong; He, Jiang | Journal of stroke and cerebrovascular diseases : the official journal of National Stroke Association | Not stated PSCI as an exclusion |
| The association between plasma soluble triggering receptor expressed on myeloid cells 2 and cognitive impairment after acute ischemic stroke | Zhu, Yinwei; Zhao, Yu; Lu, Yaling; Fang, Chongquan; Zhang, Qi; Zhang, Jintao; Ju, Zhong; Zhang, Yonghong; Xu, Tan; Zhong, Chongke | Journal of affective disorders | Not stated PSCI as an exclusion |
| Soluble ST2 and risk of cognitive impairment after acute ischemic stroke: a prospective observational study | Zhu, Yinwei; Fang, Chongquan; Zhang, Qi; Lu, Yaling; Zhang, Rui; Wang, Aili; Bu, Xiaoqing; Zhang, Jintao; Ju, Zhong; Zhang, Yonghong; Xu, Tan; Zhong, Chongke | BMC Geriatrics | Not stated PSCI as an exclusion |
| Prevalent stroke, age of its onset, and post-stroke lifestyle in relation to dementia: A prospective cohort study | Zhong, Wansi; Chen, Hui; Gong, Xiaoxian; Tong, Lusha; Xu, Xin; Zong, Geng; Yuan, Changzheng; Lou, Min | Alzheimer's & dementia : the journal of the Alzheimer's Association | Wrong outcome |
| Relation between sleep disorders and post-stroke cognitive impairment | Zhang, Yajing; Xia, Xiaoshuang; Zhang, Ting; Zhang, Chao; Liu, Ran; Yang, Yun; Liu, Shuling; Li, Xin; Yue, Wei | Frontiers in Aging Neuroscience | Wrong outcome |
| Predictive value of serum adiponectin and hemoglobin levels for vascular cognitive impairment in ischemic stroke patients | Z., Li; M., Zhu; C., Meng; H., Lin; L., Huang | Pakistan Journal of Medical Sciences | Single component |
| Plasma sDPP4 (Soluble Dipeptidyl Peptidase-4) and Cognitive Impairment After Noncardioembolic Acute Ischemic Stroke | You, Shoujiang; Bi, Yucong; Miao, Mengyuan; Bao, Anran; Du, Jigang; Xu, Tan; Liu, Chun-Feng; Zhang, Yonghong; He, Jiang; Cao, Yongjun; Zhong, Chongke | Stroke | Not stated PSCI as an exclusion |
| Association of Cardiac Biomarkers in Combination With Cognitive Impairment After Acute Ischemic Stroke | Yang, Pinni; Wang, Shuyao; Zhong, Chongke; Yin, Jieyun; Yang, Jingyuan; Wang, Aili; Xu, Tan; Zhang, Yonghong | Journal of the American Heart Association | Wrong outcome |
| Lower glomerular filtration rate after mild stroke induces cognitive impairment by causing endothelial dysfunction | Yan, Xu; Chen, Huan; Shang, Xiuli | Scientific reports | Single component |
| Research on Diagnostic Markers for Post-Stroke Cognitive Impairment | Xu, Min; Yang, Lanqing; Zhong, Zhe; Ye, Min | Alternative therapies in health and medicine | Not stated PSCI as an exclusion |
| Physical activities attenuate the negative cognitive impact from white matter hyperintensities in stroke and TIA patients with low education | Wong, Adrian; Yiu, Stanley; Lam, Bonnie Yin Ka; Leung, Kam Tat; Shi, Lin; Lo, Eugene; Abrigo, Jill; Au, Lisa Wc; Lau, Alexander; Mok, Vincent | International journal of geriatric psychiatry | Wrong outcome |
| Strategic infarct locations for post-stroke cognitive impairment: a pooled analysis of individual patient data from 12 acute ischaemic stroke cohorts | Weaver, Nick A; Kuijf, Hugo J; Aben, Hugo P; Abrigo, Jill; Bae, Hee-Joon; Barbay, Melanie; Best, Jonathan G; Bordet, Regis; Chappell, Francesca M; Chen, Christopher P L H; Dondaine, Thibaut; van der Giessen, Ruben S; Godefroy, Olivier; Gyanwali, Bibek; Hamilton, Olivia K L; Hilal, Saima; Huenges Wajer, Irene M C; Kang, Yeonwook; Kappelle, L Jaap; Kim, Beom Joon; Kohler, Sebastian; de Kort, Paul L M; Koudstaal, Peter J; Kuchcinski, Gregory; Lam, Bonnie Y K; Lee, Byung-Chul; Lee, Keon-Joo; Lim, Jae-Sung; Lopes, Renaud; Makin, Stephen D J; Mendyk, Anne-Marie; Mok, Vincent C T; Oh, Mi Sun; van Oostenbrugge, Robert J; Roussel, Martine; Shi, Lin; Staals, Julie; Del C Valdes-Hernandez, Maria; Venketasubramanian, Narayanaswamy; Verhey, Frans R J; Wardlaw, Joanna M; Werring, David J; Xin, Xu; Yu, Kyung-Ho; van Zandvoort, Martine J E; Zhao, Lei; Biesbroek, J Matthijs; Biessels, Geert Jan | The Lancet. Neurology | Wrong outcome |
| The effects of blood pressure on post stroke cognitive impairment: BP and PSCI | Wang, Yue; Li, Shiping; Pan, Yuesong; Wang, Mengxing; Liao, Xiaoling; Shi, Jiong; Wang, Yongjun | Journal of clinical hypertension (Greenwich, Conn.) | Wrong outcome |
| The potential predictive value of salivary cortisol on the occurrence of secondary cognitive impairment after ischemic stroke | Wang, Jin; Guan, Qiaobing; Sheng, Yongjia; Yang, Yi; Guo, Li; Li, Wenyan; Gu, Yanling; Han, Chenyang | Neurosurgical review | Single component |
| A New Nomogram Model for Individualized Prediction of Cognitive Impairment in Patients with Acute Ischemic Stroke | Tang, Anqi; Liu, Sanjiao; Wang, Zhi; Shao, Han; Cai, Xiuying; Li, Tan | Journal of stroke and cerebrovascular diseases : the official journal of National Stroke Association | Not stated PSCI as an exclusion |
| The association between serum uric acid level and the risk of cognitive impairment after ischemic stroke | Sun, Jing; Lv, Xinhuang; Gao, Xinxin; Chen, Zewei; Wei, Dianhui; Ling, Yi; Zhang, Junmei; Gu, Qilu; Liu, Jiaming; Chen, Weian; Liu, Suzhi | Neuroscience letters | Wrong outcome |
| Characterization and individual-level prediction of cognitive state in the first year after 'mild' stroke | Saa, Juan Pablo; Tse, Tamara; Koh, Gerald Choon-Huat; Yap, Philip; Baum, Carolyn M; Uribe-Rivera, David E; Windecker, Saras M; Ma, Henry; Davis, Stephen M; Donnan, Geoffrey A; Carey, Leeanne M | PloS one | Wrong outcome |
| The Impact of Vascular Risk Factors on Post-stroke Cognitive Impairment: The Nor-COAST Study | S., Aam; M.N., Gynnild; R., Munthe-Kaas; I., Saltvedt; S., Lydersen; A.-B., Knapskog; H., Ihle-Hansen; H., Ellekjaer; R.S., Eldholm; B., Fure | Frontiers in Neurology | Wrong outcome |
| Early Post-stroke Cognition: In-hospital Predictors and the Association With Functional Outcome | R., Sharma; D., Mallick; R.H., Llinas; E.B., Marsh | Frontiers in Neurology | Not stated PSCI as an exclusion |
| Is frailty index a better predictor than modified rankin scale for neurocognitive outcomes three months post-stroke? | R., Munthe-Kaas; S., Aam; I., Saltvedt; T., Wyller; S.T., Pendlebury; S., Lydersen; G., Hagberg; T., Schellhorn; S., Rostoft; H., Ihle-Hansen | European Stroke Journal | Abstract only |
| Prediction of Long-term Cognitive Function After Minor Stroke Using Functional Connectivity | R., Lopes; C., Bournonville; G., Kuchcinski; T., Dondaine; A.-M., Mendyk; R., Viard; J.-P., Pruvo; H., Henon; M.K., Georgakis; M., Duering; M., Dichgans; C., Cordonnier; X., Leclerc; R., Bordet | Neurology | Wrong outcome |
| Plasma Endostatin Levels at Acute Phase of Ischemic Stroke Are Associated with Post-Stroke Cognitive Impairment | Qian, Sifan; Li, Ruyi; Zhang, Chenhuan; Zhang, Rui; Guo, Daoxia; Bu, Xiaoqing; Wang, Aili; Peng, Hao; Chen, Jing; Zhang, Yonghong; He, Jiang; Xu, Tan; Zhong, Chongke | Neurotoxicity research | Not stated PSCI as an exclusion |
| Poststroke Delirium Clinical Motor Subtypes: The PRospective Observational POLIsh Study (PROPOLIS) | Pasinska, Paulina; Kowalska, Katarzyna; Klimiec, Elzbieta; Wilk, Aleksander; Szyper-Maciejowska, Aleksandra; Dziedzic, Tomasz; Klimkowicz-Mrowiec, Aleksandra | The Journal of neuropsychiatry and clinical neurosciences | Wrong outcome |
| Development of a clinical score, PANDA, to predict delirium in stroke care unit | Nakamizo, Tomoki; Kanda, Toshie; Kudo, Yosuke; Sugawara, Eriko; Hashimoto, Erina; Okazaki, Ayana; Usuda, Makoto; Nagai, Toru; Hara, Hiroshi; Johkura, Ken | Journal of the neurological sciences | Not stated PSCI as an exclusion |
| Is Frailty Index a better predictor than pre-stroke modified Rankin Scale for neurocognitive outcomes 3-months post-stroke? | Munthe-Kaas, Ragnhild; Aam, Stina; Saltvedt, Ingvild; Wyller, Torgeir Bruun; Pendlebury, Sarah T; Lydersen, Stian; Hagberg, Guri; Schellhorn, Till; Rostoft, Siri; Ihle-Hansen, Hege | BMC geriatrics | Not stated PSCI as an exclusion |
| Low Hemoglobin Levels at Admission Are Independently Associated with Cognitive Impairment after Ischemic Stroke: a Multicenter, Population-Based Study | Meng, Fanxia; Zhang, Shixin; Yu, Jie; Chen, Yi; Luo, Lunjie; He, Fangping; Wei, Ruili; Yuan, Huaiwu; Ji, Renjie; Chen, Hanfeng; Luo, Benyan | Translational stroke research | Wrong outcome |
| Risk factors for post-cerebral infarction cognitive dysfunction in older adults: a retrospective study | Ma, Fanyuan; Zhang, Qian; Li, Jinke; Wu, Liping; Zhang, Hua | BMC neurology | Single component |
| Post-stroke Cognition at 1 and 3 Years Is Influenced by the Location of White Matter Hyperintensities in Patients With Lacunar Stroke | M.D.C., Valdes Hernandez; T., Grimsley-Moore; F.M., Chappell; M.J., Thrippleton; P.A., Armitage; E., Sakka; S., Makin; J.M., Wardlaw | Frontiers in Neurology | Wrong outcome |
| Low Serum Superoxide Dismutase Is Associated With a High Risk of Cognitive Impairment After Mild Acute Ischemic Stroke | M.-S., Zhang; J.-H., Liang; M.-J., Yang; Y.-R., Ren; D.-H., Cheng; Q.-H., Wu; Y., He; J., Yin | Frontiers in Aging Neuroscience | Wrong outcome |
| Short-term Montreal Cognitive Assessment predicts functional outcome after endovascular therapy | M., Zhang; K., Wang; L., Xie; X., Pan | Frontiers in Aging Neuroscience | Single component |
| Correlation between risk factors of cognitive dysfunction and blood pressure variability after acute ischemic stroke in northwest Shanghai | M., Sun; Z., Chen; G., Li; Y., Weng; Y., Hou | The International journal of neuroscience | Wrong outcome |
| Predictive value of serum matrix metalloproteinase 9 combined with tissue inhibitor of metalloproteinase 1 for post-stroke cognitive impairment | M., Pu; Y., You; X., Wang | Journal of Clinical Neuroscience | Single component |
| Methylation of the rin3 promoter is associated with transient ischemic stroke/mild ischemic stroke with early cognitive impairment | M., Miao; F., Yuan; X., Ma; H., Yang; X., Gao; Z., Zhu; J., Bi | Neuropsychiatric Disease and Treatment | Not stated PSCI as an exclusion |
| Machine learning-based prediction of post-stroke cognitive status using electroencephalography-derived brain network attributes | Lee, Minwoo; Hong, Yuseong; An, Sungsik; Park, Ukeob; Shin, Jaekang; Lee, Jeongjae; Oh, Mi Sun; Lee, Byung-Chul; Yu, Kyung-Ho; Lim, Jae-Sung; Kang, Seung Wan | Frontiers in Aging Neuroscience | Wrong outcome |
| Machine learning-based prediction of clinical outcomes after first-ever ischemic stroke | L., Fast; U., Temuulen; K., Villringer; A., Kufner; H.F., Ali; E., Siebert; S., Huo; S.K., Piper; P.S., Sperber; T., Liman; M., Endres; K., Ritter | Frontiers in Neurology | Not stated PSCI as an exclusion |
| Prevalence and predictors of post-stroke cognitive impairment among stroke survivors in Uganda | Kaddumukasa, Martin N; Kaddumukasa, Mark; Katabira, Elly; Sewankambo, Nelson; Namujju, Lillian D; Goldstein, Larry B | BMC neurology | Wrong outcome |
| Cognition in the First Year After a Minor Stroke, Transient Ischemic Attack, or Mimic Event and the Role of Vascular Risk Factors | K., Nicolas; C., Levi; T.-J., Evans; P.T., Michie; P., Magin; D., Quain; A., Bivard; F., Karayanidis | Frontiers in Neurology | Wrong outcome |
| Could neutrophil-to-lymphocyte ratio (NLR) serve as a potential marker for delirium prediction in patients with acute ischemic stroke? a prospective observational study | K., Kotfis; M., Bott-Olejnik; A., Szylinska; I., Rotter | Journal of Clinical Medicine | Included in previous review |
| Vascular and neurodegerative markers for the prediction of poststroke cognitive impairment-results from the tabasco study | J., Molad; H., Hallevi; A., Korczyn; E., Kliper; E., Auriel; N., Bornstein; E., Ben Assayag | European Stroke Journal | Abstract only |
| PREDICTION OF COGNITIVE RECOVERY AFTER STROKE (PROCRAS): THE VALUE OF DIFFUSION-WEIGHTED IMAGING BASED MEASURES OF BRAIN CONNECTIVITY | H.P., Aben; L., De Munter; Y.D., Reijmer; J.M., Spikman; J.M.A., Visser-Meily; G.J., Biessels; P.L.M., De Kort | European Stroke Journal | Abstract only |
| Understanding and Predicting Cognitive Improvement of Young Adults in Ischemic Stroke Rehabilitation Therapy | H.B., Martinez; K., Cisek; A., Garcia-Rudolph; J.D., Kelleher; A., Hines | Frontiers in Neurology | Not stated PSCI as an exclusion |
| Severity of Lesions Involving the Cortical Cholinergic Pathways May Be Associated With Cognitive Impairment in Subacute Ischemic Stroke | H.-H., Zhong; J.-F., Qu; W.-M., Xiao; Y.-K., Chen; Y.-L., Liu; Z.-Q., Wu; D.-H., Qiu; W.-C., Liang | Frontiers in Neurology | Wrong outcome |
| Explainable machine learning for predicting neurological outcome in hemorrhagic and ischemic stroke patients in critical care | H., Wei; X., Huang; Y., Zhang; G., Jiang; R., Ding; M., Deng; L., Wei; H., Yuan | Frontiers in Neurology | Not stated PSCI as an exclusion |
| Patterns of atrial fibrillation, relevant cardiac structural and functional changes predict functional and cognitive outcomes in patients with ischemic stroke and atrial fibrillation | Guo, Jiahuan; Wang, Dandan; Jia, Jiaokun; Zhang, Jia; Liu, Yanfang; Lu, Jingjing; Tian, Ying; Zhao, Xingquan | International journal of cardiology | Wrong outcome |
| Serum tissue inhibitor of metalloproteinase-1 and risk of cognitive impairment after acute ischaemic stroke | Ge, Jinzhuo; Li, Ruyi; Yuan, Pengcheng; Che, Bizhong; Bu, Xiaoqing; Shao, Hancheng; Xu, Tan; Ju, Zhong; Zhang, Jintao; Zhang, Yonghong; Zhong, Chongke | Journal of cellular and molecular medicine | Wrong outcome |
| Post-stroke cognitive impairment remains highly prevalent and disabling despite state-of-the-art stroke treatment | Gallucci, Laura; Sperber, Christoph; Guggisberg, Adrian G; Kaller, Christoph P; Heldner, Mirjam R; Monsch, Andreas U; Hakim, Arsany; Silimon, Norbert; Fischer, Urs; Arnold, Marcel; Umarova, Roza M | International journal of stroke : official journal of the International Stroke Society | Wrong outcome |
| Risk factors and protective factors for cognitive outcomes after cerebral stroke: the results of statistical modeling using clinical data and neuroimaging | G.A., Bulyakova; L.R., Akhmadeeva; I.A., Lakman; D.E., Baikov; M.B., Isoeva; M.T., Ganieva | Arterial Hypertension (Russian Federation) | Not stated PSCI as an exclusion |
| Predictors of post-stroke delirium incidence and duration: Results of a prospective observational study using high-frequency delirium screening | Fleischmann, Robert; Andrasch, Tina; Warwas, Sina; Kunz, Rhina; Gross, Stefan; Witt, Carl; Ruhnau, Johanna; Vogelgesang, Antje; Ulm, Lena; Mengel, Annerose; von Sarnowski, Bettina | International journal of stroke : official journal of the International Stroke Society | Wrong outcome |
| A High Neutrophil-to-Lymphocyte Ratio Predicts Higher Risk of Poststroke Cognitive Impairment: Development and Validation of a Clinical Prediction Model | F., Zha; J., Zhao; C., Chen; X., Ji; M., Li; Y., Wu; L., Yao | Frontiers in Neurology | Wrong outcome |
| Transient cognitive impairment in the acute phase of stroke - prevalence, risk factors and influence on long-term prognosis in population of patients with stroke (research study - part of the PROPOLIS study) | Dros, Jakub; Kowalska, Katarzyna; Pasinska, Paulina; Klimkowicz-Mrowiec, Aleksandra | BMC neurology | Wrong outcome |
| Development and validation of a clinical model (DREAM-LDL) for post-stroke cognitive impairment at 6 months | Dong, Yi; Ding, Mengyuan; Cui, Mei; Fang, Min; Gong, Li; Xu, Zhuojun; Zhang, Yue; Wang, Xiuzhe; Xu, Xiaofeng; Liu, Xueyuan; Li, Gang; Zhao, Yuwu; Dong, Qiang | Aging | Not stated PSCI as an exclusion |
| Plasma neuropeptide Y and cognitive impairment after acute ischemic stroke | Dong, Wenjing; Lu, Yaling; Zhai, Yujia; Bi, Yucong; Peng, Yanbo; Ju, Zhong; Xu, Tan; Zhong, Xiaoyan; Zhang, Yonghong; Zhong, Chongke | Journal of affective disorders | Not stated PSCI as an exclusion |
| Predictors of Cognitive Impairment After Stroke: A Prospective Stroke Cohort Study | Ding, Meng-Yuan; Xu, Yi; Wang, Ying-Zhe; Li, Pei-Xi; Mao, Yi-Ting; Yu, Jin-Tai; Cui, Mei; Dong, Qiang | Journal of Alzheimer's disease : JAD | Included in previous review |
| The Clock Drawing Test as a predictor of cognitive decline in non-demented stroke patients | Cova, Ilaria; Mele, Francesco; Zerini, Federica; Maggiore, Laura; Rosa, Silvia; Cucumo, Valentina; Brambilla, Michela; Nicotra, Alessia; Maestri, Giorgia; Bertora, Pierluigi; Pomati, Simone; Pantoni, Leonardo | Journal of neurology | Wrong outcome |
| Systemic immune-inflammation index upon admission correlates to post-stroke cognitive impairment in patients with acute ischemic stroke | Cheng, Yongqing; Zhu, Honghong; Liu, Changxia; Li, Lei; Lin, Fangjia; Guo, Yan; Gu, Cong; Sun, Dingming; Gao, Yang; He, Guojun; Sun, Shifu; Xue, Shouru | Aging | Single component |
| Racial/Ethnic Disparities in the Alzheimer's Disease Link with Cardio and Cerebrovascular Diseases, Based on Hawaii Medicare Data | C., Siriwardhana; E., Carrazana; K., Liow; J.J., Chen | Journal of Alzheimer's Disease Reports | Wrong outcome |
| Factors associated with cognitive impairment at 3, 6, and 12 months after the first stroke among Lebanese survivors | Boutros, Celina F; Khazaal, Walaa; Taliani, Maram; Sadier, Najwane Said; Salameh, Pascale; Hosseini, Hassan | Brain and behavior | Wrong outcome |
| Network impact score is an independent predictor of post-stroke cognitive impairment: A multicenter cohort study in 2341 patients with acute ischemic stroke | Biesbroek, J Matthijs; Weaver, Nick A; Aben, Hugo P; Kuijf, Hugo J; Abrigo, Jill; Bae, Hee-Joon; Barbay, Melanie; Best, Jonathan G; Bordet, Regis; Chappell, Francesca M; Chen, Christopher P L H; Dondaine, Thibaut; van der Giessen, Ruben S; Godefroy, Olivier; Gyanwali, Bibek; Hamilton, Olivia K L; Hilal, Saima; Huenges Wajer, Irene M C; Kang, Yeonwook; Kappelle, L Jaap; Kim, Beom Joon; Kohler, Sebastian; de Kort, Paul L M; Koudstaal, Peter J; Kuchcinski, Gregory; Lam, Bonnie Y K; Lee, Byung-Chul; Lee, Keon-Joo; Lim, Jae-Sung; Lopes, Renaud; Makin, Stephen D J; Mendyk, Anne-Marie; Mok, Vincent C T; Oh, Mi Sun; van Oostenbrugge, Robert J; Roussel, Martine; Shi, Lin; Staals, Julie; Valdes-Hernandez, Maria Del C; Venketasubramanian, Narayanaswamy; Verhey, Frans R J; Wardlaw, Joanna M; Werring, David J; Xin, Xu; Yu, Kyung-Ho; van Zandvoort, Martine J E; Zhao, Lei; Biessels, Geert Jan | NeuroImage. Clinical | Wrong outcome |
| The Impact of Covert Lacunar Infarcts and White Matter Hyperintensities on Cognitive and Motor Outcomes After Stroke | Auriat, Angela M; Ferris, Jennifer K; Peters, Sue; Ramirez, Joel; Black, Sandra E; Jacova, Claudia; Boyd, Lara A | Journal of stroke and cerebrovascular diseases : the official journal of National Stroke Association | Wrong outcome |
| Incidence and predictors of post-stroke cognitive impairment among patients admitted with first stroke at tertiary hospitals in Dodoma, Tanzania: A prospective cohort study | Alphonce, Baraka; Meda, John; Nyundo, Azan | PloS one | Wrong outcome |
| Predictive Value of the Alberta Stroke Program Early CT Score (ASPECTS) in the Outcome of the Acute Ischemic Stroke and Its Correlation with Stroke Subtypes, NIHSS, and Cognitive Impairment | A., Esmael; M., Elsherief; K., Eltoukhy | Stroke Research and Treatment | Wrong outcome |
